# Nanocellulose-based adsorbents for water remediation: role of surface functionalisation and isocyanate chemistry

**DOI:** 10.1039/d6na00619a

**Published:** 2026-10-09

**Authors:** Yerkebulan Altynov, Dana Ashiraliyeva, Bibigul Rakhimova, Aidyn Kazhmuratov, Altynbek Kazez, Kalampyr Bexeitova, Seitkhan Azat, Kenes Kudaibergenov

**Affiliations:** a Satbayev University, Department of Materials Science, Nanotechnology and Engineering Physics 22 Satbayev Street Almaty 050013 Kazakhstan k.kudaibergenov@satbayev.university; b Kazakh National Women's Teacher Training University, Department of Chemistry 114/8 Gogol Street Almaty 050000 Kazakhstan; c Laboratory of Engineering Profile, Satbayev University 22 Satbayev Street Almaty 050013 Kazakhstan

## Abstract

Water contamination by synthetic dyes, heavy metals, and emerging micropollutants has intensified the search for sustainable, high-performance adsorbents. Nanocellulose (NC) – available as cellulose nanocrystals, nanofibrils, and bacterial nanocellulose – has become a leading bio-based platform for water remediation owing to its high specific surface area, abundant surface hydroxyl groups, and amenability to chemical modification. The performance of NC sorbents is governed largely by surface functionalisation, yet existing reviews treat the available modification chemistries unevenly. In particular, isocyanate (carbamation) chemistry is almost invariably reduced to a single entry in a list of modification methods, despite being one of the few strategies able to combine, within a single platform, covalent crosslinking, tunable surface hydrophobicity, and the introduction of orthogonal reactive handles for ligand conjugation. This review addresses that gap. After surveying NC types, preparation routes, target pollutants, and adsorbent architectures (hydrogels, membranes, and aerogels), it provides a critical, water-remediation-focused analysis of NC isocyanate chemistry: the carbamation mechanism and competing side reactions, the reactivity of aromatic *versus* aliphatic isocyanates, site-selectivity in TDI and IPDI, structure–property relationships, documented applications in oil–water separation, dye removal, and heavy-metal sorption, and the role of isocyanates as molecular bridges in multifunctional composites. The toxicological profiles of diisocyanates and emerging non-isocyanate polyurethane (NIPU) alternatives are critically assessed, and the principal research gaps and priorities are identified.

## Abstract 2

Isocyanate chemistry offers a versatile, high-capacity route to functionalise nanocellulose adsorbents for water remediation—systematised here for the first time. Image created in BioRender.

## Graphical abstract

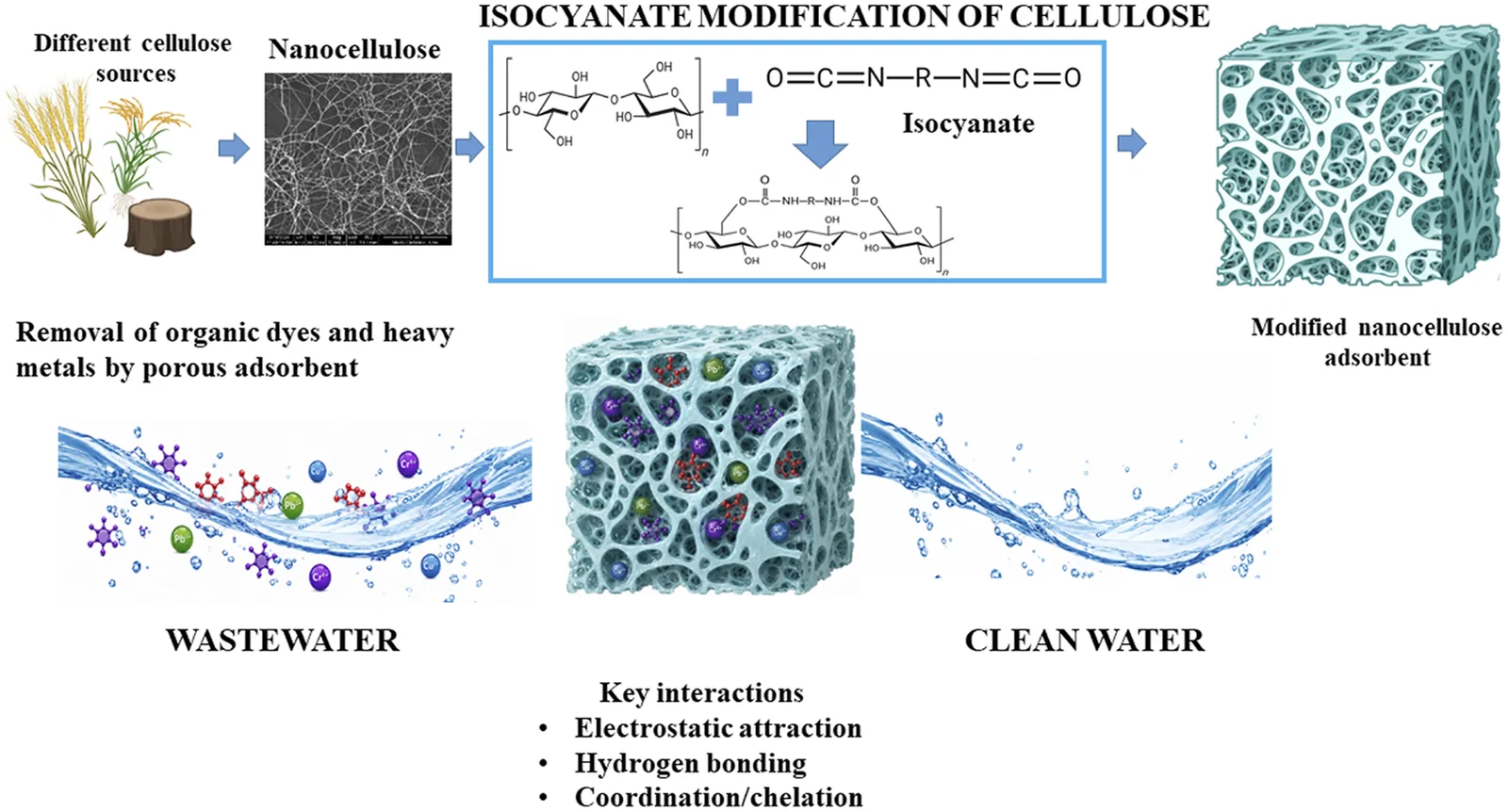

## 1. Introduction

The rapid growth of industrial sectors directly contributes to the large-scale release of harmful substances into the environment.^1–3^ According to recent statistics, approximately 267.5 billion cubic metres of wastewater are polluted with domestic and industrial waste every year. Of this volume, only 63% (168.8 billion cubic metres) is collected for treatment, but in practice, only 146.3 billion cubic meters actually undergoes the treatment process.^4^

The main contaminants of concern are synthetic organic dyes and heavy metal ions. Both resist biological degradation and remain toxic at concentrations of a few milligrams per litre^5–7^ (Section 5). Traditional technologies such as coagulation, flocculation, electrochemical treatment, membrane technology, photocatalysis, and biological treatment have limitations in terms of their ability to remove specific pollutants. These technologies require expensive equipment and consume large amounts of energy.^5,8^ Therefore, researchers are focusing on developing new, environmentally friendly, efficient, and cost-effective adsorbent materials.

One of the most promising areas of research is the use of biopolymer materials derived from renewable natural resources. Nanocellulose is of particular interest, as it has unique physicochemical properties, such as a high specific surface area, a porous structure, and numerous reactive functional groups on its surface.

Cellulose, one of the most abundant organic polymers on Earth, is characterised by its biodegradability, high mechanical strength, and chemical stability.^9^

Isocyanates, specifically toluene diisocyanate (TDI), 4,4′-diphenylmethane diisocyanate (MDI), and hexamethylene diisocyanate (HDI), are widely used in the modification of nanocellulose.^10–12^

Nanocellulose can be produced from agricultural and industrial waste materials, such as rice and soybean hulls. This process enhances the sustainability and resource efficiency of the technology.^13^ Thus, nanocellulose becomes not only an adsorbent but also an element of a strategy for valorising agricultural waste and reducing the environmental burden.^13,14^

Despite the breadth of this activity, the available reviews treat the various surface-modification chemistries unevenly: isocyanates are almost always mentioned only as a single item in a list of modification methods, typically reduced to the statement that they form urethane bonds, without mechanistic analysis, structure–property discussion, or comparison with alternative functionalisation strategies. This gap is significant, because isocyanate-based carbamation is one of the few NC modification routes that can simultaneously deliver covalent crosslinking, tunable surface hydrophobicity, and orthogonal reactive handles for subsequent ligand conjugation – properties of direct relevance to the design of robust, selective sorbents for flow-through water treatment.

The aim of this review paper is to systematically analyse the various types of functionalised nanocellulose-based adsorbents, their methods of production and modification, and their effectiveness in removing organic and inorganic contaminants from water. Research gaps and future directions are also identified.

The present review is distinguished from existing surveys by placing isocyanate chemistry at its centre. To the best of the authors' knowledge, it is the first review to provide a critical, water-remediation-focused treatment of NC isocyanate chemistry, encompassing the carbamation mechanism and its side reactions, the structure–property relationships of isocyanate-modified NC, documented and prospective applications in pollutant removal, the use of isocyanates as molecular bridges in multifunctional composites, and the toxicological profile of diisocyanates together with emerging non-isocyanate polyurethane (NIPU) alternatives (Section 4). The surrounding sections on nanocellulose types and preparation (Sections 2 and 3), target pollutants (Section 5), and adsorbent architectures (Section 6) provide the context within which this contribution is situated. They are deliberately condensed, since each has been comprehensively surveyed elsewhere,^15–20^ and the critical treatment is concentrated in Section 4.

## 2. Nanocellulose: types and methods of obtaining

### 2.1. Types of nanocellulose

Nanocellulose is a biopolymeric nanomaterial derived from plant biomass; it is characterised by high mechanical strength, a high specific surface area, and low density.^13^ Depending on the production method, morphology, and structure, there are several main types of nanocellulose materials. The most common ones are cellulose nanocrystals (CNC), cellulose nanofibrils (CNF), bacterial nanocellulose (BNC).^3,21,22^ These materials differ in their origin, structure, degree of crystallinity, mechanical properties, and applications (Table 1). However, they all share the main properties of cellulose, such as biodegradability and the ability to be chemically modified.^3,21,22^

**Table 1:** Types of nanocellulose and their main properties

Type of nanocellulose	Alternative titles	Typical dimensions	Main properties and structure	Natural sources	Reference
CNC	Nanocrystalline cellulose	Diameter: 3–70 nm, length: 100–2000 nm	High crystallinity (>70%), rigid rod-like structure, high tensile strength	Wood, cotton, flax, agricultural waste	23–25
CNF	Microfibrillated cellulose (MFC)	Diameter: 2–100 nm, length: several micrometres	Long, flexible fibres containing amorphous and crystalline regions (moderate crystallinity), high viscosity, and water retention capacity	Wood pulp, plant fibres (sugarcane, hemp)	23 and 26–28
BNC	Biocellulose, microbial cellulose	Diameter: 20–100 nm	Exceptional purity (free of lignin or hemicellulose), high porosity, high degree of polymerisation, and a 3D network structure	Synthesised by bacteria (for example, *Acetobacter xylinum*)	23, 26 and 29

CNC are a type of material that has a unique structure. It consists of glucose units that are linked together by linear β-1,4-glycosidic bonds. The structure of CNC has alternating crystalline and amorphous regions, which gives it a low density and large specific surface area. CNC also have reactive surface groups that enhance their potential for use in a variety of functional materials. These materials include adsorption systems, which are used to remove pollutants from water and air. The reactive surface groups on CNC make them ideal for this purpose, as they can bind to pollutants and remove them from the environment.^30^ In addition, the high level of crystallinity in CNC ensures their mechanical strength and stability. CNC are often produced through acid hydrolysis using strong mineral acids, such as sulfuric, hydrochloric, phosphoric, and nitric acid. This process dissolves the amorphous areas, while preserving the crystalline areas.^31^ As a result, highly crystalline solid rod-like particles are formed; during H_2_SO_4_ hydrolysis (60 °C, 60 min), their length ranges from 30 to 100 nm.^31^

Cellulose nanofibrils have a long and flexible fibrous structure with a length greater than 1 µm and a cross-sectional area of approximately 5 nm. This structure gives them a high aspect ratio, which enhances their ability to form complex spatial network structures.^32^ CNF is primarily obtained by “top-down” mechanical defibrillation methods.^33^ A typical approach is a two-stage mechanical treatment consisting of refining and high-pressure homogenisation.^33^ Long, flexible nanofibrils with a diameter of ∼10–100 nm and a length-to-diameter ratio exceeding 50–100 are formed.^34^ To facilitate the dispersion of fibrillae, TEMPO oxidation is performed beforehand. This method introduces carboxylate groups onto the surface of the fibrillae, weakens the inter-fibrillar bonds, and improves the separation of the nanofibrillae.^33^

BNC is synthesised by some microorganisms, mainly acetic bacteria of the genus *Komagataeibacter*.^35^ Bacterial cellulose is a unique and pure material. It does not contain lignin or hemicellulose, and its fibrils are extremely thin (less than 100 nm) and highly crystalline (approximately 84–89%).^36^ BNC forms a three-dimensional nanofibrillar network with high porosity and water retention. This is why it is widely used in medicine for wound healing.^35,36^

### 2.2. Methods of nanocellulose preparation

Methods of nanocellulose production are classified into chemical, mechanical, and biological approaches; their advantages and disadvantages are compared in Table 2.

**Table 2:** Comparative analysis of nanocellulose production methods

Method	Process description	Advantages	Disadvantages	Reference
Acid hydrolysis	Treatment with strong acids (H_2_SO_4_, HCl) to remove amorphous regions	High reaction rate; CNC with high crystallinity can be easily obtained	Equipment corrosion; issues related to acid waste disposal	21, 26 and 28
Mechanical treatment	High-pressure homogenisation, ultrasonication, ball milling	Environmentally friendly; does not require aggressive chemicals	High energy consumption; non-uniform fibre length	26 and 37
TEMPO oxidation	Selective oxidation of C_6_ hydroxyl groups to carboxyl groups	Facilitates fibrillation; enables formation of stable CNF dispersions	High cost of catalysts (TEMPO)	26 and 28
Enzymatic hydrolysis	Use of cellulase enzymes to break down bonds	Mild conditions; environmentally friendly; preserves fibre structure	Longer reaction time and lower rate compared to chemical methods	26 and 28

Acid hydrolysis is the most widely used method for producing CNC.^31^ The raw material is treated with concentrated acids (H_2_SO_4_ and HCl), which hydrolyse the amorphous regions of cellulose. This process results in the release of rod-like nanoparticles with high crystallinity and stability.^31^ During H_2_SO_4_ hydrolysis, sulfate ester groups (–OSO_3_H) are formed on the CNC surface, giving it a negative charge and helping to stabilise the suspension.

Alkaline treatment is often used as a preliminary step to remove lignin and hemicellulose.^38^ Boiling in a concentrated NaOH solution helps to soften the structure of the cellulose-based raw material, making it easier to access reagents during subsequent processing steps.^38^

TEMPO-mediated oxidation is a chemical technique used to introduce functional groups into molecules.^33^ The TEMPO system oxidises the primary hydroxyl groups at the C6 position of glucopyranose units to carboxyl groups. These carboxylates introduce surface charges, which enhance electrostatic repulsion between fibrils and significantly facilitate subsequent mechanical defibrillation.^33^

Ultrasonication disrupts hydrogen bonds through acoustic cavitation (10–100 kJ mol^−1^), while steam explosion disrupts the cell-wall structure by rapid pressure release after brief heating under pressure; both serve as auxiliary pre-treatments.^38^ The use of agricultural by-products, such as rice husks, soybean hulls, and seed coats, as a source for nanocellulose production holds great promise.^21^ For example, CNC, derived from rice husk after multiple chemical treatment stages, shows an increase in crystallinity from 22.63% to 67.16%. The average particle diameter is 19 ± 3.3 nm, and the length is 195 ± 24 nm.^25^ Agricultural residues are typically burned, but their use in nanocellulose production enables efficient use of biomass and reduces the environmental impact.^21^ A generalised technological scheme for producing nanocellulose from agricultural waste is presented in Fig. 1.

**Fig. 1:**
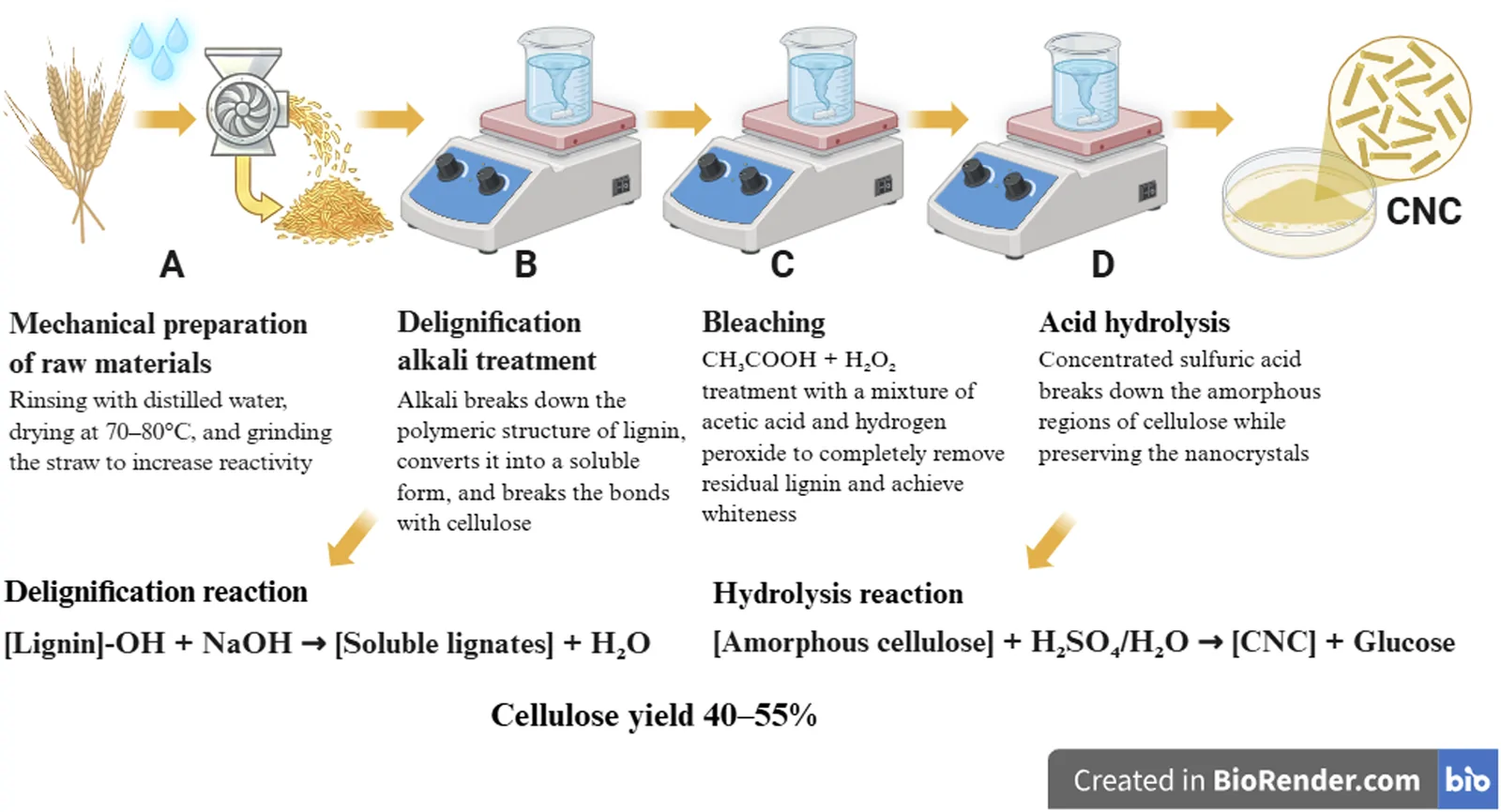
Schematic representation of nanocellulose production from agricultural waste, including pretreatment, extraction methods, and the final nanocellulose product (Created in BioRender).

## 3. Surface modification of nanocellulose

### 3.1. General principles of modification

The surface modification of nanocellulose through its hydroxyl groups greatly enhances the functional properties of the material.^39,40^ Reactive –OH groups permit the introduction of a range of chemical functionalities onto the surface of nanocellulose through relatively simple chemical reactions, including oxidation, acetylation, and other methods of functionalisation.^39,40^

Unlike bulk cellulose, nanocellulose modification is predominantly carried out under heterogeneous conditions. This means that reactions mainly occur on the surface of the material.^41,42^ A significant proportion of hydroxyl groups in the nanocellulose structure are located at the surface. Their content (excluding less reactive C3 hydroxyl groups) can be up to approximately 15%.^41,42^ The high number of surface hydroxyl groups in nanocellulose makes it particularly promising for the development of functional materials. The main modification approaches and their purposes are summarised in Table 3.

**Table 3:** Principal methods for the surface modification of nanocellulose

Modification type	Chemical reaction	Introduced groups	Purpose	Reference
TEMPO oxidation	Selective oxidation of C_6_–OH → –COOH	–COO^−^ (carboxylate)	Increase surface charge; improve CNF dispersion	43
Sulfation	During H_2_SO_4_ hydrolysis	–OSO_3_H (sulfate ester)	Negative surface charge; suspension stability	44
Isocyanate modification	–OH + R–NCO → urethane linkage	Urethane (–NHCOO–)	Hydrophobicity; compatibility with polymer matrices	45 and 46
Polymer grafting (“grafting”)	Initiation *via* surface –OH groups	Polymer chains (*e.g.*, PVA, PA)	Improved compatibility with composite matrices	47 and 48
Amination	Introduction of amine groups	–NH_2_	Binding of anionic pollutants	49
Thiol modification	Introduction of thiol groups	–SH	Chelation of mercury and heavy metals	50

Beyond these direct modification routes, nanocellulose also serves as a support for molecularly imprinted polymers, which achieve selectivity through templated recognition cavities rather than through surface chemistry; several synthesis techniques have been developed for this purpose, including bulk, precipitation, emulsion and suspension polymerisation, and surface imprinting.^51,52^ This approach lies outside the scope of the present review. Of the routes listed in Table 3, diisocyanates are distinctive in enabling controlled two-stage functionalization, in which one NCO group forms a urethane linkage while the second remains available for further reaction; this bifunctionality is the basis of the two-stage grafting strategies analysed in Section 4. A recent review provides a comprehensive survey of these and related surface-modification strategies, including esterification, etherification, click chemistry and isocyanate-based urethanisation.^53^

## 4. Isocyanate chemistry of nanocellulose: a critical review

Although nanocellulose (NC) has emerged over the past decade as one of the most intensively studied bio-based platforms for water remediation, the role of isocyanate chemistry in governing its surface and bulk properties remains underrepresented in the specialist review literature. A survey of seven representative reviews on NC-based sorbents and water treatment materials published between 2020 and 2026 reveals a consistent pattern. In all seven works – Tshikovhi *et al.* (2020),^54^ Salama *et al.* (2021),^16^ Iqbal *et al.* (2022),^17^ Norizan *et al.* (2022),^15^ Aoudi *et al.* (2022),^18^ Abdelhamid (2024),^19^ and Das *et al.* (2022)^20^ – isocyanates are mentioned only as one item in a list of NC modification methods, often reduced to a single declarative statement such as “isocyanates form urethane bonds”,^46^ with no mechanistic analysis, no structure–property discussion, and no comparison with alternative functionalisation strategies. The only review devoted specifically to this topic – Abushammala and Mao (2019, Molecules)^45^ – was published in 2019, is framed within the context of nanocomposite materials science, and focuses primarily on nanocomposite applications, with water purification addressed as one of several potential application areas. A recent review of CNC surface chemistry, published independently of the present analysis, which likewise concludes that the urethanisation approach remains underutilised for surface tuning.^27^

Isocyanate functionalisation is one of the few NC modification strategies capable of simultaneously addressing four material design objectives: (i) covalent crosslinking, imparting wet-state stability and mechanical integrity; (ii) surface hydrophobisation, tunable across a wide range from moderate wettability (water contact angle ∼90°) to near-superhydrophobic behaviour; (iii) controlled introduction of orthogonal reactive handles – pendant secondary isocyanates, amines, urea, or thiol groups – available for subsequent conjugation with a broad range of nucleophilic ligands; and (iv) interfacial crosslinking between NC and a polyurethane matrix, with direct consequences for the architecture of foams, sponges, and membranes in flow-through filtration systems (Fig. 2 and Table 4).

**Fig. 2:**
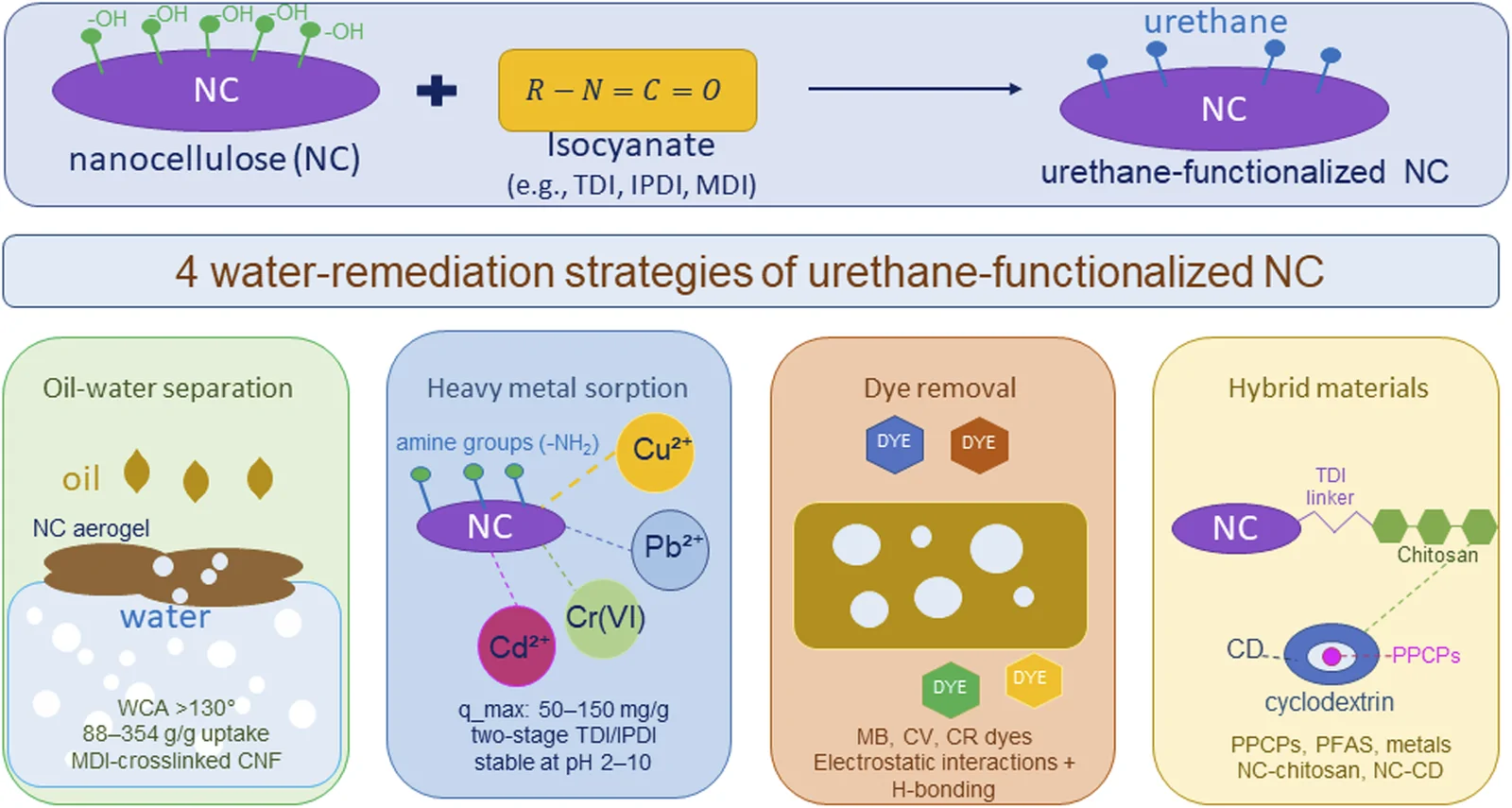
Isocyanate chemistry of nanocellulose and four water-remediation strategies: oil–water separation, heavy metal sorption, dye removal, and hybrid sorbents for PPCPs and PFAS.

**Table 4:** Summary of research on the modification of nanocellulose *via* mono- and diisocyanates

Category	CNC/CNF	Isocyanate	Matrix/chemical system	Reference
Functional nanocellulose	CNC	3,5-Dimethylphenyl isocyanate	—	55
	CNC	*n*-Octadecyl isocyanate (ODI)	—	56
	CNC	TDI	Photodegradable polymer	57
Improvement of nanocellulose properties	CNF	*n*-Octadecyl isocyanate (ODI)	—	58
	CNC	TDI	Castor oil	59
	CNC	TDI	Poly(hydroxybutyrate-*co*-valerate) (PHBV)	60
	CNF	HDI	Alkyl diamines	61
	CNF	HDI	—	62
Treating nanocellulose with non-polar matrices	CNC, CNF	Isophorone diisocyanate (IPDI)	Polycaprolactone	63 and 64
	CNC	IPDI	Polybutylene adipate-terephthalate	65 and 66
	CNC	TDI	Polylactide	67
Cross-linking of the nanocellulose matrix	CNC	MDI	Known polyols	68
	CNC	HDI	Polyurethane	69
	CNF	Polymeric methylene diphenyl diisocyanate	Lignin–soy polyol + polymeric methylene diphenyl diisocyanate	70
	CNC	Isophorone diisocyanate	Isophorone diisocyanate + trifunctional polyether alcohol	48

### 4.1. Mechanism and reaction chemistry

#### 4.1.1. The carbamation reaction

The isocyanate group –N=C=O is among the most electrophilic heterocumulenes employed in synthetic polymer chemistry.^55^ Its reactivity originates from the electron-deficient central carbon atom, which is readily attacked by nucleophilic species – in particular, the surface hydroxyl groups located at the C2, C3, and C6 positions of the anhydroglucose units (AGU) of nanocellulose.^45^ The resulting addition reaction yields a urethane (carbamate) linkage of the general form NC–O–C(=O)–NH–R without release of any small-molecule by-products:NC–OH + R–N=C=O → NC–O–C(=O)–NH–R

By strict green chemistry metrics, this reaction is fully atom-economical – no leaving groups, by-products, or salt waste are generated – which differentiates it from esterification with acid chlorides (releasing HCl), anhydrides (releasing free acids), or activated esters (requiring carbodiimide coupling agents). For NC-based sorbents intended for water treatment this clean stoichiometry confers a practical advantage: residual reagents and decomposition products capable of migrating into the treated water are inherently minimised.

The principal practical difficulty arises from the competing reaction of isocyanates with water. Isocyanate hydrolysis yields an unstable carbamic acid, which rapidly decomposes to a primary amine and CO_2_; the resulting amine then attacks a second isocyanate molecule, forming a urea linkage. Because NC is typically produced and stored as an aqueous suspension, this side reaction becomes the default pathway unless specifically suppressed. The standard solution is solvent exchange of the aqueous NC suspension into an anhydrous medium – acetone, DMF, toluene, or DMSO – prior to isocyanate addition.^44,45^ This requirement makes the isocyanate protocol more demanding to execute and more difficult to scale than aqueous TEMPO-oxidation or moisture-tolerant silylation.

#### 4.1.2. Reactivity: aromatic *vs.* aliphatic isocyanates

The intrinsic reactivity of an isocyanate towards nanocellulose depends strongly on the electronic environment of the N=C=O group, which is governed by the nature of the substituent R. Aromatic isocyanates – including phenyl isocyanate (PI), TDI, and MDI – are more electrophilic than their aliphatic analogues: conjugation with the aromatic ring withdraws electron density from the nitrogen atom, leaving the central carbon more electron-deficient, and additionally stabilises the negative charge developing on nitrogen in the transition state.^45^ As a result, aromatic isocyanates are generally one to two orders of magnitude more reactive towards aliphatic alcohols than their aliphatic counterparts.^45^ In the context of NC modification, this reactivity hierarchy translates into pronounced practical differences. Aromatic isocyanates typically react with surface hydroxyls within a few hours at moderate temperatures (60–80 °C) without requiring a catalyst. This facilitates rapid functionalisation but increases the likelihood of competing side reactions with trace moisture and with functional groups already present on the NC surface; bulky MDI is additionally subject to steric hindrance at the crystalline NC surface. Aliphatic isocyanates, by contrast, generally require either a catalyst – typically dibutyltin dilaurate (DBTDL) or a tertiary amine such as triethylamine (TEA) or 1,4-diazabicyclo[2.2.2]octane (DABCO) – or elevated temperatures and extended reaction times.^45^ These additional requirements bring improved selectivity, lower volatility, and substantially reduced occupational toxicity. This requirement is not absolute, however: it depends on the viscosity of the reaction medium and the functionality of the reagent as much as on the electronic character of the isocyanate. A polyfunctional aliphatic isocyanate such as an HDI biuret or trimer can react with cellulose hydroxyls at elevated temperature without any catalyst or solvent, since its higher functionality and the low viscosity it affords the reaction medium compensate for the lower intrinsic electrophilicity of the aliphatic NCO group.^71^

#### 4.1.3. Site-selectivity: the *para*/*ortho* effect in TDI

Among the diisocyanates used in NC chemistry, 2,4-toluene diisocyanate is distinctive because its two NCO groups are non-equivalent and react at substantially different rates.^46,48^ The *para*-isocyanate (*p*-NCO), positioned diagonally across the ring from the methyl substituent, is both electronically activated and sterically unhindered; the *ortho*-isocyanate (*o*-NCO), adjacent to the methyl group, is subject to steric crowding and mild inductive deactivation. The *para* group has been reported to be approximately five to ten times more reactive towards aliphatic alcohols at room temperature than the *ortho* group.^45,46^

This molecular asymmetry enables a two-stage modification strategy. In the first stage, conducted under kinetic control, the *para*-isocyanate reacts preferentially with the surface hydroxyls of NC, yielding an NC-*p*-carbamate with a pendant *o*-NCO; in the second stage, the *o*-NCO is reacted with a chosen nucleophilic ligand – an amine, thiol, alcohol, or polymer end-group – to graft the desired functional group *via* a secondary urethane or urea linkage.^46,48^

The selectivity achieved in practice is, however, often below the kinetic ideal. Abushammala (2019)^46^ demonstrated quantitatively that a significant fraction of TDI molecules react through both isocyanate groups with NC surface hydroxyls, yielding a dysfunctional product devoid of a free reactive group for subsequent conjugation. A related principle has been demonstrated for ionic crosslinking of carboxylated CNF: the concentration of the nanocellulose dispersion at the moment a bi- or multifunctional agent is introduced determines whether it forms predominantly intra-particle or inter-particle linkages, with dilute dispersions favouring reaction within a single fibril element.^72^ This principle was established for a different chemistry, but it applies directly to TDI grafting. There, both NCO groups reacting with the same NC surface is treated as an unwanted side reaction rather than as a parameter that could be controlled. To address this, an analytical method was developed based on hydrolysis of residual *o*-NCO groups in acidified DMSO to primary amines, followed by quantification by pH titration.^73^ Under optimised conditions (35 °C, TDI/CNC molar ratio of 3), *para*/*ortho* selectivity reached 93% – a threefold improvement over conventional conditions (75 °C, ratio of 1).^46^ This quantitatively defined platform underpins the rational design of all TDI-modified NC sorbents discussed below.

#### 4.1.4. Competing side reactions and their implications for sorbent design

Beyond hydrolysis, several additional side reaction pathways must be considered. Primary and secondary amines – whether introduced deliberately in the second stage of the TDI protocol or generated by partial hydrolysis of residual NCO groups – react with isocyanates two to three orders of magnitude faster than alcohols, yielding urea linkages.^42,54^ As a result, amine functionality is consumed before it can participate in metal ion coordination or hydrogen bonding with organic micropollutants. A further complication arises for TEMPO-oxidised NC, whose surface sodium carboxylate groups are, in principle, susceptible to the well-established condensation of carboxylate salts with aryl isocyanates, which proceeds *via* a mixed carboxylic–carbamic anhydride intermediate and extrudes CO_2_ to yield an amide bond.^74^ Although this reaction has been characterised under controlled organic-synthesis conditions rather than on cellulose surfaces, its occurrence would complicate the infrared interpretation of TDI- or MDI-modified TOCNF, since the resulting amide carbonyl absorption overlaps with the urethane and urea bands already present. To ensure reproducibility across studies, quantitative reporting of residual NCO content, urea formation, and total nitrogen content is recommended as standard practice in publications on isocyanate-modified NC sorbents.

### 4.2. Structure–property relationships in isocyanate-modified NC

Isocyanate modification simultaneously alters several physicochemical properties of nanocellulose. The relationships between molecular-level modification parameters – isocyanate type, degree of substitution, and grafting geometry – and the macroscopic properties relevant to sorbent performance are not systematically discussed within a unified framework. The following subsections systematise the available data across five descriptors: surface wettability, thermal stability, mechanical properties and porosity, degree of substitution, and crystallinity.

#### 4.2.1. Wettability and crystallinity

Unmodified NC is strongly hydrophilic: water contact angles (WCA) for CNC and TEMPO-oxidised CNF films range from 17° to 35°, depending on surface charge density, biomass source, and preparation method.^17^ Carbamation systematically increases the WCA, with the magnitude of the change governed by the chain length and chemical nature of the R-group. In a systematic study,^75^ a homologous series of *n*-alkanols (C2–C8) was grafted onto CNC using TDI as a linker: the WCA increased monotonically from 32° ± 2 for the unmodified material to 35° (C2), 52° ± 3 (C4), 104° ± 1 (C6), and 120° ± 5 for the C8 variant, consistent with the progressive increase in surface alkyl chain density. A one-step alternative – direct reaction with *n*-octadecyl isocyanate (OI, C_18_H_37_–NCO) – yields hydrophobic NC without the need for a TDI bridging step.^63^

For oil–water separation applications, a WCA above 130–140° is sufficient for self-cleaning behaviour and high oil selectivity, and this regime is achievable by isocyanate grafting. Superhydrophobicity (WCA > 150°), however, additionally requires hierarchical surface roughness, which in NC systems is generated by assembly into freeze-dried aerogels or by vapour-phase deposition of fluorinated silanes; isocyanate chemistry alone cannot achieve this regime without structural support.^17^

X-ray diffractograms of TDI-, MDI-, HDI-, and OI-modified CNC consistently retain the principal cellulose Iβ reflections at 2*θ* ≈ 14.8°, 16.6°, and 22.7°.^45,46^ The magnitude of the accompanying decrease in crystallinity index depends strongly on the degree of substitution achieved. Modification protocols yielding low surface DS retain crystallinity within a few percent of the unmodified precursor, whereas more extensive grafting can be considerably more disruptive: a monoisocyanate reaction reaching a surface DS of 0.40 has been reported to reduce crystallinity from 91.4% to 76.5%, a decrease of some 16%.^76^ This contrasts with fully homogeneous silane-mediated functionalisation routes, which can eliminate the crystalline cellulose I signature almost entirely.^73^ The heterogeneous, surface-confined nature of isocyanate modification, rather than any intrinsic mildness of the chemistry itself, is therefore what limits crystallinity loss. A related contrast holds for TEMPO-mediated oxidation, which retains the cellulose I structure even as surface hydroxyls are converted to carboxylate groups, in distinction to carboxymethylation, which typically proceeds *via* mercerising conditions and converts the crystal form to cellulose II.^77^

The underlying mechanism is as follows: under anhydrous conditions and at temperatures below 90 °C, isocyanates react exclusively with surface-accessible hydroxyl groups. The extent to which the internal hydrogen-bond network remains intact depends on how much of the accessible surface is derivatised.^45^ Retaining crystallinity preserves the high aspect ratio that underpins the network-forming capacity of NC and its effective specific surface area. This is itself a trade-off, however, rather than an unqualified benefit: a more crystalline, less disrupted particle also presents fewer internally accessible sites for subsequent functionalisation or sorption.^78^ The same surface confinement that protects crystallinity therefore also limits the density of sites available for ligand grafting or for the developing hydrated pore network.

#### 4.2.2 Thermal stability

Carbamation substantially improves the thermal stability of NC, which is important both for high-temperature regeneration of saturated sorbents and for processing NC into composite matrices. The urethane bond is thermally stable up to 200–250 °C; aromatic urea linkages in diisocyanate-crosslinked networks are more stable still, with an onset decomposition temperature of approximately 280–300 °C.^45^ A particularly notable effect is observed at the level of char yield: Jiang and Hsieh (2017)^79^ reported that MDI-crosslinked CNF aerogels retained 43% of their mass as carbonaceous residue at 500 °C, compared with only 9.1% for the uncrosslinked counterpart, a result attributed to suppression of volatile-releasing decomposition pathways by the covalent network. This picture, however, requires three qualifications that are rarely addressed together in the isocyanate-modified NC literature. First, part of any measured thermal-stability gain for CNC in particular reflects removal of sulfate ester groups introduced during acid hydrolysis, which catalyse cellulose degradation at lower temperatures; substitution of these groups during modification therefore contributes to the apparent improvement independently of the urethane linkage itself. Second, sample handling can confound the comparison: a control experiment isolating the effect of post-reaction washing alone found the onset decomposition temperature of an unmodified sample dropping from 246 °C to 214 °C after washing, so that the true gain attributable to isocyanate modification of a properly washed control was 38 °C rather than the 6 °C apparent when the modified sample is compared only with the untreated, unwashed starting material.^80^ Third, nanoscale size itself lowers thermal stability relative to bulk cellulose: onset decomposition has been reported to fall from 260 °C for bulk cellulose to 200 °C for TEMPO-oxidised CNF and to 116 °C for sulfated CNC, a reduction of 60–145 °C attributed to higher surface-to-volume ratio and to the destabilising effect of the surface functional groups themselves.^78^ Reported thermal-stability improvements upon isocyanate modification should therefore be read as net effects layered on top of these confounding factors, rather than as a clean measure of urethane-bond stability alone.

Competing surface chemistries, moreover, are not necessarily inferior on this metric. Cellulose modified with azetidinium salts has been reported to show an onset-temperature increase of roughly 100 °C, and a silane–polyol grafting route has been reported to shift the midpoint of degradation by around 140 °C^73,81^ – both larger than the gains typically reported for isocyanate carbamation. Within isocyanate chemistry itself, the crosslinking agent matters: isocyanurate rings formed from HDI trimer degrade at substantially higher temperature (onset ≈474 °C) than the urethane linkages they accompany (≈388 °C), so that thermal performance depends on which linkage type dominates the network rather than on the presence of urethane bonds *per se*.^71^

Finally, thermal characterisation of isocyanate-modified NC sorbents is, without exception, performed on the unloaded material. This is a significant omission for sorbents intended for regeneration by thermal desorption, since the presence of a bound metal cation can alter both the onset temperature and the mechanism of decomposition: sorbents loaded with different cations have been shown to degrade *via* distinct pathways and at shifted temperatures relative to the unloaded material, in one case introducing an additional decomposition event not present before metal binding.^78^ Thermal stability data for isocyanate-modified NC sorbents in the metal-loaded state are entirely absent from the literature reviewed here, and we identify this as a further gap.

#### 4.2.3. Mechanical properties, porosity and degree of substitution

Diisocyanate crosslinking converts the hydrogen-bonded inter-particle contact network of NC into a covalently linked network with qualitatively different mechanical behaviour. In the MDI–CNF aerogel system reported in ref. 82, the optimally crosslinked aerogel exhibited a nearly threefold increase in specific surface area to 228 m^2^ g^−1^ and a doubling of pore volume to ∼1 cm^3^ g^−1^, accompanied by the formation of mesopores in the 9–12 nm range. The compressive response of the crosslinked network is cooperative rather than destructive through inter-particle slip, as observed in physically gelled or freeze-dried NC aerogels. Two consequences follow directly for sorbent design. First, the increase in specific surface area directly expands the number of accessible adsorption sites – the primary parameter governing sorption capacity in flow-through configurations. Second, mechanical robustness enables column and pressurised operation: uncrosslinked NC aerogels collapse under moderate hydrostatic pressure or during repeated saturation–regeneration cycles, whereas crosslinked analogues retain their porous architecture over many cycles. Such a combination of properties is not straightforwardly achieved through alternative crosslinking chemistries – citric acid, glutaraldehyde, or polyamide-epichlorohydrin – without compromising other performance parameters.

These structural gains must, however, be weighed against two counteracting consequences of carbamation that bear directly on aqueous-phase adsorption: the consumption of surface hydroxyl groups and the increase in surface hydrophobicity.

The loss of hydroxyl functionality is quantitatively modest. Surface substitution in TDI-carbamated CNC typically reaches only 5–10% of the accessible hydroxyl groups, corresponding to 1.0–1.5 mmol of urethane linkages per gram,^46^ so that 90–95% of the surface hydroxyls remain unreacted. Two compensating effects operate in the opposite direction. First, the urethane linkage is not chemically inert with respect to adsorption: the N–H proton and the carbonyl oxygen act as a hydrogen-bond donor–acceptor pair, a role documented for urethane groups in polyurethane-based sorbents.^83,84^ Second, in two-stage protocols the pendant NCO group is converted into an amine, urea or thiol functionality of higher metal-binding affinity than the hydroxyl group it displaced.

The hydrophobicity increase is the more consequential trade-off, and its sign depends on the target pollutant: advantageous for oil–water separation (Section 4.2.1), but a liability for aqueous ionic pollutants, since it impedes wetting of the pore network.

This is compounded by a methodological caveat. The BET surface area of nanocellulose is not an absolute quantity: it depends on the probe molecule and measurement temperature, differing by roughly 25% between N_2_ at −196 °C and *n*-octane at 25 °C for the same specimen.^85^ Reported areas for nanocellulose sorbents vary even more widely depending on the method used—from around 12 m^2^ g^−1^ by BET to 400–430 m^2^ g^−1^ estimated from cationic demand.^86–88^ That such discrepancies correspond to real differences in accessible sorption sites, rather than measurement artefacts alone, is shown directly for dense TEMPO-CNF nanopapers: increasing grammage from 5 to 20 g m^−2^ raised adsorbed mass from 150 to only 330 mg m^−2^, well below the 600 mg m^−2^ expected if internal groups contributed as effectively as surface ones, consistent with double-layer overlap rendering much of the internal charge inaccessible.^89^ The threefold BET increase reported above for the MDI–CNF system should therefore not be assumed to translate proportionally into accessible sorption capacity.

Consistent with this, mechanical robustness through covalent crosslinking has repeatedly proven necessary rather than merely beneficial: aerogels assembled from uncrosslinked CNF or TEMPO-CNF have been reported to collapse or lose their functional architecture on handling, whereas covalently crosslinked analogues retain both shape and porosity.^88,90,91^ The stakes of neglecting this are illustrated starkly in a packed-column configuration, where uncrosslinked TEMPO-CNF swelled into a gel and blocked flow entirely, a failure resolved only by partial esterification to reduce water retention at the cost of some carboxyl functionality.^88^ Neither the restriction of substitution degree nor the confinement of hydrophobic groups to an outer shell has been systematically examined for isocyanate-modified NC, and we identify this as a further gap.

The degree of substitution (DS) is the parameter most directly under the chemist's control: it governs hydrophobicity, dispersibility in organic solvents, the mechanical strength of crosslinked architectures, and, in two-stage protocols, the number of active sites available for target pollutant binding. For TDI-carbamated CNC, surface substitution typically reaches 5–10% of the available hydroxyl groups, corresponding to approximately 1.0–1.5 mmol of urethane bonds per gram of CNC.^46^ The optimised reaction yielded a DS(NCO) of 7.2 ± 0.3% with a free isocyanate loading of 1.33 ± 0.07 mmol g^−1^ (ref. 46 and 92) – values that are ultimately limited by the specific surface area of the crystalline particle.

This value should not, however, be read as an intrinsic ceiling for isocyanate grafting on CNC: it reflects the outcome of a specific protocol rather than a limit of the chemistry itself. Comparative studies of surface DS achieved with different isocyanate reagents span a considerably wider range, from 0.07 for direct reaction with a long-chain monoisocyanate to 0.59–0.96 under extended reaction conditions and up to 1.4 for IPDI grafting under DBTDL catalysis with a large reagent excess.^76^ This upper value approaches the theoretical maximum of 1.5 surface hydroxyls per two anhydroglucose units set by the crystalline structure of CNC. The comparatively modest loading of 1.33 mmol g^−1^ obtained under the standard TDI protocol therefore represents one point within an accessible range rather than a hard upper bound. Higher loadings are attainable at the cost of harsher conditions, larger reagent excess, or reduced crystallinity (Section 4.2.1).

This loading nonetheless remains the effective ceiling for ligand density in two-stage grafting protocols conducted under standard conditions. It also compares favourably with competing surface-modification routes: a silane–polyol grafting approach achieves only 0.32 mmol g^−1^ of grafted polyol under comparable heterogeneous conditions,^73^ roughly a quarter of the loading available through isocyanate chemistry. Translating this ceiling into a metal-binding capacity requires care regarding stoichiometry. Ion-exchange studies on carboxylated TEMPO-oxidised cellulose show that divalent cations such as Pb^2+^ and Ca^2+^ bind surface carboxylate groups with a 1 : 1 molar stoichiometry rather than the 2 : 1 ratio that simple valence considerations might suggest.^77^ If this stoichiometry extends to amine-terminated isocyanate-grafted ligands, the maximum theoretical loading of divalent metal cations is correspondingly higher than a 2 : 1 assumption would predict, and the practical adsorption capacity is correspondingly more sensitive to whatever fraction of the grafted sites remains sterically accessible in the hydrated state.

### 4.3. Isocyanate-modified nanocellulose for water remediation

Despite the methodological maturity of NC isocyanate chemistry, its targeted application to pollutant removal from water remains scarce in the literature, in contrast to the substantially larger body of literature on silane-, TEMPO-, or periodate-modified NC sorbents. A systematic survey of the available work reveals three distinct strategies, each exploiting a fundamentally different aspect of urethane chemistry and each retaining substantial unrealised potential. Additional tools are drawn from materials science studies originally targeting other applications but directly relevant to water treatment upon deliberate reinterpretation (strategy d). The four strategies are discussed in turn, followed by identification of three persistent gaps that define the priorities for future research.

#### 4.3.1. Oil–water separation and composite foam applications

A key study in this area concerns MDI-crosslinked CNF aerogels.^82^ The authors implemented a process scheme that circumvents the principal obstacle of isocyanate chemistry – incompatibility with aqueous media. CNF hydrogels were first formed by freeze–thaw cycling, then solvent-exchanged into acetone, in which the reaction with MDI was carried out directly; the acetone was subsequently exchanged for *tert*-butanol prior to freeze-drying.^82^ The resulting aerogel exhibited a combination of properties unattainable by single-step modification: a WCA exceeding 130° without additional vapour-phase treatment, a nearly threefold increase in specific surface area to 228 m^2^ g^−1^, complete gravitational separation of chloroform–water mixtures, and repeatable elastic recovery under compression.^82^ In contrast to multistep silane- or carboxylic acid-based alternatives,^93^ MDI simultaneously achieves covalent crosslinking, porosity enhancement, and surface hydrophobisation within a single reaction step. Quantitative uptake capacities for individual oils were not reported in that study; for context, comparable hydrophobic NC aerogels achieve 88–143 g g^−1^ for diesel fuel and up to 354 g g^−1^ for chloroform.^94,95^ The primary advantage of MDI crosslinking is mechanical regenerability rather than maximum single-cycle absorption capacity.

The second strategy differs fundamentally from the first: here the isocyanate does not selectively modify NC but instead polymerises *in situ* with a polyol to form a three-dimensional polyurethane network in which NC acts as a functional filler. This architecture draws on industrially established polyurethane chemistry, making the approach suitable for transfer into commercial filtration systems. The resulting sorbents integrate heterogeneous functional sites – NC carboxylates, polyurethane urethane nodes, and any additional functional groups introduced through the polyol – into a single mechanically robust structure. An analogous approach has been demonstrated in bio-based polyurethane nanocomposites prepared from plant-derived polyols and aliphatic diisocyanates for the removal of methylene blue and crystal violet.^96^

This architecture also invites direct comparison with silane-mediated compatibilisation strategies pursued for the same underlying problem: dispersing a hydrophilic cellulosic nanomaterial within a hydrophobic polyurethane matrix. Silanisation followed by grafting of a bio-based polyol onto CNC in a homogeneous reaction medium has been reported to yield composite polyurethane foams with up to a fivefold increase in compressive strength relative to foams reinforced with unmodified or heterogeneously modified CNC,^73^ indicating that the silane–polyol route is a serious competitor to isocyanate-mediated NC incorporation for mechanical reinforcement of the resulting foam, even though it addresses matrix compatibilisation rather than functional site density.

#### 4.3.2. Heavy metal sorption *via* the two-stage TDI/IPDI route

The third strategy makes most direct use of the bifunctional character of diisocyanates: one NCO group reacts with the surface hydroxyls of NC while the second remains free for conjugation with a chelating ligand – a primary amine, polyamine, thiol, or iminodiacetate.

This strategy has been most systematically implemented in Abushammala and Mao.^46,92^ Following TDI grafting onto CNC and hydrolysis of residual *ortho*-NCO groups in acidified DMSO (see Section 4.1.3), primary aromatic amines are formed, covalently anchored to the NC surface through a tolylene–urethane linker, at a loading of 1.33 ± 0.07 mmol g^−1^ with 93% selectivity^46,92^

This loading is directly comparable to the amine density achieved by silane grafting: APTES-modified microfibrillated cellulose has been reported to carry 2.3 mmol g^−1^ of amine groups, some 1.7-fold higher than the TDI route, and to adsorb Ni(ii), Cu(ii) and Cd(ii) at 160, 200 and 472 mg g^−1^ respectively^97^ – substantially higher than earlier estimates for silane-modified NC sorbents. The two routes differ, however, in the hydrolytic stability of the surface linkage under conditions relevant to real effluents. The siloxane (Si–O–C) bond connecting APTES to the cellulose surface is not stable under alkaline conditions: nitrogen leaching from APTES-modified microfibrillated cellulose has been shown to increase from around 240 to 1700 µg L^−1^ as pH rises from 6 to 9, consistent with base-catalysed hydrolysis of the siloxane linkage.^97^ The tolylene–urethane linker of the TDI route is not subject to this failure mode, which may be an advantage for sorbents intended for alkaline process streams, though its own stability across the full pH range relevant to water treatment has not been separately established.

Whether TDI- or IPDI-mediated polyamine grafting on NC would approach the capacities reported for polyamine-functionalised cellulose sorbents more broadly remains untested. The available data indicate that capacity scales with nitrogen donor density rather than falling within a fixed range: cellulose derivatives bearing two, three or four grafted amino groups reach a maximum capacity of 249.7 mg g^−1^ for Cd(ii) and 401.2 mg g^−1^ for Pb(ii) at a nitrogen content of 5.2 mmol g^−1^,^98^ while a nanocellulose-supported polyamine system has achieved up to 952 mg g^−1^ for Cu(ii).^99^ No study has yet grafted a polyamine onto NC *via* the TDI/IPDI route and measured the resulting capacity, so it is not possible to say where such a system would fall on this scale; the free NCO loading of 1.33 mmol g^−1^ established above sets an upper bound on the amine density attainable in a single grafting step, which is lower than the 5.2 mmol g^−1^ nitrogen content of the highest-capacity comparator system.

An analogous gap exists for thiol ligands: TDI/IPDI-mediated grafting of cysteine or mercaptopropionic acid onto CNC or CNF has not yet been experimentally demonstrated for heavy-metal sorption, although a related isothiocyanate route to thiocarbamate-linked, sulfur-donor cellulose derivatives for metal chelation has been explored,^53^ suggesting that the underlying chemistry is accessible even though it has not been applied *via* the two-stage isocyanate protocol discussed here.

#### 4.3.3. Adjacent studies and research gaps

A number of isocyanate-NC studies originally developed for other purposes carry direct relevance to water treatment. First, investigations of OI-modified CNF aimed at improving NC dispersibility in nonpolar matrices^63^ yielded materials with WCA values of 113–119° and high hydrophobic layer stability – properties relevant to oil–water separation upon subsequent structural assembly into an aerogel or membrane. Second, HDI-crosslinked CNF networks studied in the context of elastomeric biocomposites are promising for microplastic sorption: the compressive elasticity of aliphatic networks is critical for sorbent regeneration during repeated cycles involving solid particles, whereas rigid MDI-based networks are unsuited to this task. The methodological toolkit for both material classes is already validated; systematic transfer into the water treatment context with accompanying sorption characterisation has not yet been undertaken.

A survey of the available literature identifies three significant gaps, each representing a priority direction for future research.

First, systematic quantitative sorption characterisation is rarely reported in isocyanate-NC sorbent studies for the applications discussed above (oil–water separation, dye removal *via* composite foams, heavy-metal sorption). One partial exception illustrates both what is possible and what remains missing: a triisocyanate-crosslinked cationic CNF aerogel has been characterised with full Langmuir isotherms, defined pH and temperature, and multi-cycle regeneration data, achieving 293, 531 and 600 µmol g^−1^ for three anionic dyes at pH 7 and 25 °C, with elution regeneration in aqueous KCl and stable performance in packed-column operation.^100^ In this system, however, the isocyanate crosslinker performs a purely structural function – stabilising the aerogel network against disintegration in water – while the sorption capacity itself is governed by cationic trimethylammonium groups introduced in a separate step; the zeta-potential characterisation supporting the proposed electrostatic mechanism was in fact obtained on the uncrosslinked precursor rather than on the crosslinked sorbent used in the uptake experiments. Isocyanate chemistry is therefore not established, even in this most fully characterised example, as the variable that controls sorption performance. For applications in which isocyanate grafting is intended to introduce or modulate the sorption-active functionality itself – as in the amine- and thiol-grafting strategies of Section 4.3.3 – no comparably complete quantitative dataset exists.

Where such data are eventually reported, four methodological safeguards, established in the wider adsorption literature but rarely applied even to well-studied NC sorbents, would substantially strengthen their interpretation. First, apparent uptake of divalent metal cations at near-neutral pH can be confounded by precipitation of insoluble hydroxides and oxides that co-separate with the sorbent on filtration; without a matrix-matched blank control lacking sorbent, a fraction of the reported capacity may reflect precipitation rather than adsorption.^101^ Second, fitting adsorption isotherms by linearised regression can introduce errors of the order of tens of percent in the derived constants and can obscure the presence of more than one type of sorption site; nonlinear regression with explicit residual analysis is the more reliable approach.^102^ Third, the maximum capacity extracted from a Langmuir fit is sensitive to the concentration range over which the isotherm was measured and is only meaningful as a saturation capacity once the isotherm has genuinely reached a plateau; reporting the initial concentration range alongside *q*_max_ is therefore essential for cross-study comparison.^103^ Fourth, the mechanism inferred from macroscopic uptake data is rarely confirmed spectroscopically; where combined X-ray photoelectron and X-ray absorption spectroscopy has been used to test a proposed binding mechanism, the resulting picture has in at least one case ruled out the mechanism initially proposed from adsorption data alone, underscoring the value of direct spectroscopic verification.^104^ A worked example combining several of these elements – nonlinear isotherm fitting with full statistical reporting and isothermal titration calorimetry for adsorption thermodynamics – exists for a PEI-grafted TEMPO-oxidised CNF sorbent, though not for an isocyanate-modified system.^105^ This contrasts with the well-developed quantitative characterisation tradition established for TEMPO-oxidised, silane-modified, and phosphorylated NC sorbents, several examples of which nonetheless share the same limitations.

Second, direct head-to-head comparisons between isocyanate-NC and silane-NC sorbents under identical experimental conditions are absent. The two fields have developed in parallel across different research groups with minimal methodological overlap, making rational selection between them for a given application impossible. Such comparisons could be achieved without new synthetic work, by benchmarking TDI–CNC–NH_2_ against APTES–CNC–NH_2_, and MDI-crosslinked CNF aerogel against MTMS-silylated CNF aerogel, across a standard set of metrics including *q*_max_, selectivity in competitive mixtures, regeneration retention, stability across pH 2–10, and residual monomer content.

Third, isocyanate-NC sorbents have not been systematically evaluated for the fastest-growing contaminant categories – microplastics, pharmaceuticals and personal care products (PPCPs), and per- and polyfluoroalkyl substances (PFAS). Urethane chemistry is theoretically well suited to these targets: fine-tuning of surface polarity through R-group selection, incorporation of cyclodextrins *via* two-stage grafting, and the mechanical robustness of crosslinked networks for flow-through configurations. Experimental work in this direction is virtually non-existent, representing a significant direction for future investigation.

The case for pursuing this direction is reinforced by the broader nanocellulose literature. A dedicated review of NC for microplastic removal reports removal efficiencies of up to 98% for unmodified and physically modified NC systems, and notes that introducing cationic surface groups onto a CNF aerogel enhances microplastic uptake roughly nine-fold relative to the unmodified aerogel.^106^ Direct experimental confirmation comes from bacterial cellulose biopolymers, which removed up to 99% of polystyrene microplastics from contaminated water and retained 93–96% efficiency in dried form, with the particles both adsorbed onto and incorporated within the three-dimensional nanofibrillar network.^107^ These findings establish both that NC is intrinsically well suited to microplastic capture and that surface functionalisation is the decisive performance lever – precisely the variable that isocyanate chemistry controls. The complete absence of any isocyanate-modified NC study in this rapidly expanding field therefore represents not a peripheral omission but a clearly defined and readily addressable research opportunity.

### 4.4. Isocyanate as a chemical bridge in multifunctional composites

In contrast to the strategies discussed in Section 4.3, where isocyanate-modified NC was directly tested for pollutant removal, the present section addresses works in which the isocyanate functions as a chemical bridge – a reactive intermediate that covalently connects NC to another functional component. This role is enabled by two properties not reproduced by silanes or anhydrides: controllable selectivity between the first and second NCO groups of a diisocyanate, and compatibility with a broad range of nucleophiles including hydroxyls, amines, thiols, carboxylates, and amides.^45^ Four practical scenarios are discussed below: TDI as a general-purpose linker, IPDI as a site-selective platform, HDI as a flexible spacer, and isocyanate as a bridge to biomolecules.

#### 4.4.1. TDI as a general-purpose linker

2,4-Toluene diisocyanate remains the most extensively studied linker for NC modification. As described in Section 4.2.1, a homologous series of *n*-alkanols (C2–C8) was grafted onto CNC *via* a two-stage TDI scheme: the *para*-NCO reacts with surface hydroxyls at 35 °C in anhydrous toluene, yielding a CNC-carbamate with a pendant *ortho*-NCO (selectivity 93% (ref. 46 and 92)), which is then reacted with the chosen alcohol. The same protocol, applied with functionalised alcohols, converts NC into a site-specifically modified sorbent; the second step is equally compatible with amines, thiols, polymer end-groups, and chelating ligands as the second-stage nucleophile.^45^ Proof of concept beyond the NC literature is provided by Anne *et al.*,^108^ who conjugated β-cyclodextrin through a bifunctional isocyanate linker and successfully removed 2,4-dinitrophenol from natural water samples; transposition of this design onto a CNC support has not yet been experimentally demonstrated.

#### 4.4.2. Aliphatic diisocyanates: IPDI and HDI

IPDI is noteworthy in that its two NCO groups differ substantially in their electronic and steric environments. The primary NCO group (–CH_2_–NCO), pendant to the cyclohexane ring, is more sterically accessible; the secondary NCO (–CH(CH_3_)–NCO), adjacent to the methyl substituent and embedded within the ring, is more hindered. Critically, the relative reactivity of the two groups depends on the choice of catalyst: in the absence of a catalyst the primary NCO is more reactive, whereas in the presence of DBTDL the selectivity inverts in favour of the secondary group – as demonstrated experimentally by Girouard *et al.*^48^ using ATR-FTIR, elemental analysis, and ^13^C NMR. Under DBTDL catalysis, the secondary NCO reacts preferentially with the surface hydroxyls of CNC, leaving the more reactive primary NCO free for conjugation with a target functional group, yielding a material with a defined free NCO group available for further functionalisation.^48^

For water treatment applications this confers two advantages. First, unlike TDI, where the maximum *para*/*ortho* selectivity is 93%,^46^ IPDI with DBTDL provides near-quantitative selectivity: the number of free NCO groups per gram of material is precisely equal to the number of attached IPDI molecules, eliminating losses of active functionality and yielding a stoichiometrically predictable material for subsequent graftingGirouard *et al.*^48^ also observed a 35 °C upward shift in the onset of thermal degradation of CNC – an advantage for thermally regenerable sorbents.

The practical limitation of the Girouard *et al.*^48^ study is that IPDI-modified CNC was tested exclusively as a filler in polyurethane composites, with no sorption testing in water. Nevertheless, the methodological maturity of the approach is sufficient to regard IPDI-CNC as a viable starting material for the development of selective NC-based chelating sorbents. A related bifunctional reagent worth noting is 3-isocyanatopropyltriethoxysilane, which carries both an isocyanate group for urethane formation and a triethoxysilyl group capable of hydrolytic condensation into a siloxane network; such a reagent can in principle couple isocyanate- and silane-based surface chemistries within a single molecule, though its application to NC modification for water treatment has not been reported.^53^ A complementary monoisocyanate strategy is illustrated by 2-isocyanatoethyl methacrylate, in which the isocyanate group reacts with surface hydroxyls to form the usual urethane linkage while the pendant methacrylate group remains available for subsequent radical polymerisation; this orthogonal reactivity has been used to graft acrylic functionality onto CNC for copolymerisation with methyl methacrylate, illustrating a route by which isocyanate chemistry can serve purely as an anchoring step for a chemically distinct second-stage functionality, in this case a polymerisable vinyl group rather than a chelating ligand.^76^

HDI provides a fundamentally different type of spacer compared to the rigid aromatic TDI and MDI: a flexible aliphatic C_6_ chain that retains segmental mobility in the hydrated state. Two consequences follow for water treatment applications. First, aliphatic HDI-crosslinked networks are promising where repeated compression cycles are required – notably for microplastic sorption – since rigid MDI-based analogues accumulate fatigue damage under such conditions. A direct comparison between MDI- and HDI-crosslinked NC aerogels under matched water-treatment conditions has not yet been reported, although a biuret trimer of an aliphatic diisocyanate has been used to crosslink a cationic CNF aerogel for aqueous dye sorption, demonstrating that HDI-type crosslinking is compatible with functional water-treatment architectures even where the crosslinker itself plays a structural rather than sorption-determining role.^100^ Second, aliphatic HDI networks have a lower glass transition temperature and higher segmental mobility than their aromatic counterparts, which may support higher water flux through membranes at equivalent sorbent site densities. HDI and IPDI are additionally substantially less volatile and less toxic than TDI and MDI – a relevant consideration in reagent selection for future material development.

#### 4.4.3. Alternative linking strategies

Blocked polyisocyanates (PICs) constitute a distinct category, in which the isocyanate groups are temporarily protected by a blocking agent – phenol, oxime, imidazole, or ε-caprolactam – that dissociates upon heating to regenerate free NCO groups *in situ*. This allows mixing directly with aqueous nanocellulose (NC) suspensions, where heating above the deblocking temperature initiates urethane crosslinking without solvent exchange, free monomer handling, or moisture sensitivity. An analogous conceptual strategy is implemented by dynamic hindered ureas (Oluz *et al.* (2024)^109^), which dissociate *in situ* into an isocyanate and an amine upon moderate heating, enabling solvent-free melt-phase grafting without handling free diisocyanate monomers. A caution applies to both of these strategies and, more broadly, to any composite in which NC is combined with a diisocyanate or a polyisocyanate matrix component: covalent linkage between NC surface hydroxyls and the deblocked or *in situ*-generated NCO groups is often assumed on the basis of the known reactivity of the two species, but is rarely confirmed directly, since spectroscopic signals from the comparatively small NC fraction are typically obscured by the surrounding matrix; a mechanical reinforcement effect is consistent with covalent incorporation of NC into the network, but does not on its own rule out predominantly physical entrapment.^71^

The isocyanate linker enables the preparation of hybrid sorbents that are not readily accessible through alternative modification chemistries. Connecting NC (hydroxyl-rich) to chitosan (amine-rich) through a diisocyanate bridge yields biodegradable hybrids with orthogonal sorption sites: hydroxyl groups coordinate hard metal cations while amine groups target soft ones. TDI-linked NC–chitosan systems of this type have not yet been experimentally demonstrated.

Strong, if indirect, support for the NC–chitosan bridge design is provided by recent work on physically assembled cellulose–chitosan aerogels: a bidirectional chitosan–CNF aerogel was reported to achieve adsorption capacities exceeding 300 mg g^−1^ toward a range of micro- and nanoplastics – polystyrene, polyethylene, polypropylene, and PMMA – while operating stably in a continuous-flow configuration.^110^ Replacement of the non-covalent assembly with a diisocyanate bridge could yield a covalently stabilised analogue with improved wet-state durability and regenerability, directly addressing the microplastic and PPCP/PFAS targets identified in Section 4.3.3.

Cyclodextrins (α-, β-, and γ-CD), as discussed in the context of Anne *et al.*,^108^ provide host–guest cavities for binding hydrophobic micropollutants; direct attachment of CD to an NC support through a diisocyanate linker could yield a sorbent combining high specific surface area, host–guest selectivity, and mechanical robustness – an approach of potential relevance to the removal of PPCPs and PFAS, which are poorly retained by conventional ion-exchange sorbents.

### 4.5. Toxicological profile and green alternatives

The toxicological dimensions of diisocyanates are directly relevant to the practical deployment of isocyanate-modified NC materials in water treatment applications and are therefore addressed systematically in this section. The following subsections cover the toxicological profile of diisocyanates, the analytical characterisation of residual monomeric NCO in final materials, emerging NIPU, and a comparative assessment of application scenarios in which isocyanate-based NC chemistry retains clear advantages over available alternatives.

#### 4.5.1. Toxicological profile and residual monomer

Diisocyanates are associated with well-documented occupational health hazards. The principal compounds used in NC modification – MDI, TDI, HDI, and IPDI – are classified as skin and respiratory sensitisers, and as irritants to the eyes, skin, and respiratory tract; MDI and TDI are additionally considered suspected carcinogens.^45^ The primary adverse effects include respiratory sensitisation and occupational asthma, which can develop through both inhalation and dermal exposure. Aromatic diisocyanates (TDI, MDI) present greater toxicological risks than aliphatic ones (HDI, IPDI) due to their higher volatility and reactivity; where isocyanate modification is employed, selection of HDI or IPDI substantially reduces the toxicological risks associated with synthesis. This distinction extends beyond occupational exposure during synthesis to the biocompatibility of the resulting cured material: polyurethane films prepared from a biobased polyol and an HDI trimer, evaluated *in vitro* against fibroblasts, supported cell viability in excess of 90%, consistent with the generally lower toxicity reported for degradation products of aliphatic isocyanates relative to their aromatic counterparts.^71^ This risk differential is reflected in the regulatory treatment of the two classes: a recent survey of cellulose surface-modification chemistry likewise identifies the toxicity and handling hazards of aromatic diisocyanates, particularly TDI and MDI, as a factor motivating regulatory scrutiny and driving interest in isocyanate-free alternatives.^53^

An important technical distinction relevant to regulatory classification is the following: a fully reacted polyurethane or NC carbamate contains no free NCO groups and does not carry the same risks as monomeric diisocyanate. The urethane (carbamate) bond formed by reaction of an NCO group with an NC hydroxyl is chemically inert, is not an allergen, and presents no toxicological hazard under normal service conditions. Regulatory restrictions on diisocyanates target specifically the free monomer in the finished product, not the chemically bound isocyanate residues in a fully reacted material. The practical difficulty, however, is that isocyanate conversion in NC material synthesis rarely reaches 100%. A fraction of the monomer may remain unreacted within the pores of the material, in inter-particle voids, or on the external surface – and this residual concentration can be substantial, particularly in syntheses employing an excess of diisocyanate, which is a standard approach for maximising the degree of substitution. Without analytical characterisation, the regulatory and toxicological status of a given material cannot be reliably established. Quantitative determination of residual NCO in NC materials can be achieved by three methods. A straightforward screening method is ATR-FTIR spectroscopy: the diagnostic NCO absorption band at ∼2270 cm^−1^ is well resolved from the cellulose bands and detectable at concentrations above ∼0.05 wt% without any sample preparation. Higher accuracy is provided by dibutylamine back-titration with hydrochloric acid, which quantifies free NCO with a precision better than 0.01 wt%. Finally, the pH-titration method described in ref. 92 specifically for NC systems simultaneously characterises free NCO content and its conversion to primary amines upon acid hydrolysis, which is particularly valuable in two-stage TDI grafting protocols. Quantitative reporting of residual NCO content is recommended as standard practice in publications on isocyanate-modified NC sorbents, analogous to the established norm of reporting residual TEMPO/NaClO in TEMPO-oxidised CNF or residual sulfate in acid-hydrolysed CNC. Without such data, cross-study comparison, regulatory classification, and prediction of monomer migration into treated water remain impossible – a consideration of particular relevance to water treatment applications.

#### 4.5.2. Green alternatives: NIPU

Growing regulatory pressure and increasing demand for sustainable materials have driven the rapid development of NIPU chemistry – a field that is virtually absent from the NC sorbent literature for water treatment and that represents a significant area for future development in bio-based sorbent research.

The fundamental NIPU reaction is the aminolysis of cyclic carbonates by primary or secondary polyamines, yielding polyhydroxyurethanes (PHU):^111^Cyclic carbonate + H_2_N–R → –O–C(=O)–NH–R + –OH

In contrast to classical polyurethane chemistry, NIPU offers several fundamental advantages directly relevant to water treatment.^111,112^ First, cyclic carbonates are not classified as sensitisers or carcinogens, which substantially reduces the occupational health risks and regulatory requirements associated with their use. Second, many NIPU reactions proceed in water or water/alcohol mixtures, enabling direct use of aqueous NC suspensions without the labour-intensive solvent exchange procedure. Third, cyclic carbonates can be produced from CO_2_*via* epoxide carbonation, making NIPU synthesis a route for carbon dioxide utilisation and conferring a carbon-negative process potential. Finally, the polyhydroxyurethane backbone retains free hydroxyl groups along the main chain, which themselves constitute additional sorption sites – a feature not present in classical urethane chemistry. Integration of NC with NIPU remains at an early stage, but a small body of key publications has accumulated over the past five years. Ge *et al.* (2021)^113^ synthesised PHU nanocomposites with nanocrystalline cellulose *via* cyclic carbonate aminolysis, demonstrating significant improvements in thermomechanical properties. The more recent work of Wijeratne *et al.* (2025)^114^ represents one of the first documented cases of incorporating CNC and chitin nanocrystals (ChNCs) into a non-isocyanate polyurethane. The authors employed vanillyl bis(cyclocarbonate) (VABC), poly(tetramethyleneoxy)diamine (PTMODA), and bis(aminomethyl)norbornane (NORB) to produce segmented thermoplastic polyhydroxyurethane nanocomposites. Small-angle X-ray scattering, ATR-FTIR, and AFM showed that partially deacetylated ChNCs were covalently grafted to PHU through aminolysis of terminal carbonate groups, whereas sulfated CNC blended physically with PHU without covalent integration – a critical observation for sorbent design, as it indicates that amine functionalisation of NC is necessary for covalent incorporation into a NIPU matrix. In parallel, a 2025 study^115^ demonstrated the feasibility of synthesising NIPU foams incorporating CNC and phytic acid as a functional filler, providing proof of concept that NIPU architectures are compatible with foam form factors relevant to water treatment applications. These three studies^64,113,115^ provide an initial methodological basis for the development of NC-NIPU sorbent systems. None of them, however, has been tested in water: all investigations focus on thermomechanical, rheological, and structural characterisation of the composites. The transfer of NIPU-NC architectures into the water treatment context represents a significant direction for future research.

One synthetic strategy that has not yet been experimentally realised warrants specific attention. TEMPO-oxidised NC, carrying C6 aldehyde or carboxylate groups, could be converted to an epoxide or cyclic carbonate derivative and subsequently incorporated into a NIPU network through aminolysis with poly- or diamines, by analogy with published work on PEI-functionalised cellulose.^105^ Suitable amine reagents include polyethylenimine, tetraethylenepentamine, and cystamine. The anticipated product would combine the mechanical attributes of a covalently crosslinked polyurethane network – wet-state stability, thermal resistance, and structural integrity through repeated regeneration cycles – with diverse sorption functionality: amine and hydroxyl groups would provide chelation sites for Cu(ii), Pb(ii), Cd(ii), Cr(vi), and Zn(ii). When bio-derived cyclic carbonates and polyamines are used, the material would be of fully renewable origin and would avoid the occupational health concerns associated with free diisocyanate monomers. No experimental publication implementing this scheme for water remediation has been identified in the literature, representing a well-defined direction for future experimental investigation.

#### 4.5.3. Alternative chemistries and outlook

Beyond NIPU, several adjacent approaches offer potential for replacing isocyanate chemistry in NC sorbent design. Dynamic hindered ureas, developed by Oluz *et al.* (2024),^109^ exploit an intramolecular isocyanate–amine equilibrium activated by heat, enabling isocyanate-like reactivity without handling free monomeric diisocyanates. Thiol–ene photochemistry on alkene-functionalised CNC provides an atom-economical and rapid route under UV irradiation with no toxicological concerns, although it requires prior introduction of vinyl or allyl groups onto the NC surface. Carbodiimide-mediated coupling between TEMPO-oxidised CNF carboxylates and amine-bearing ligands yields chemically stable amide bonds without toxic reagents. Finally, Schiff base reactions between dialdehyde cellulose, prepared by periodate oxidation, and polyamines produce imine linkages that can be selectively reduced to stable secondary amines using NaBH_4_ or NaBH_3_CN. Each of these approaches carries a distinct profile of advantages and limitations; however, all avoid the occupational hazards associated with handling free diisocyanate monomers.

This gap is narrower than it might first appear for two of the four alternatives discussed above. Schiff base chemistry between dialdehyde cellulose and a chitosan derivative has been used to prepare a crosslinked aerogel with a measured Langmuir capacity of some 785 mg g^−1^ for methylene blue, together with defined pH dependence and multi-cycle regeneration data.^116^ A related acetal-forming route – condensation of dialdehyde cellulose fibrils with amyloid nanofibrils rather than reduction to a stable amine – has yielded an aerogel with quantified Langmuir capacities for both dyes and Pb(ii), and with a selectivity coefficient for Pb(ii) in the presence of Ca(ii) and Mg(ii) more than two orders of magnitude higher than for the background cations.^117^ A third, chemically distinct alternative – covalent coupling of dialdehyde cellulose nanofibrils to lignin nanoparticles *via* condensation rather than imine formation – has been shown to increase the wet tensile strength of the resulting composite film roughly tenfold relative to the unmodified precursor, from below 3 MPa to over 30 MPa, demonstrating that covalent, isocyanate-free crosslinking of NC can independently deliver the wet-state mechanical stability that isocyanate carbamation is otherwise relied upon to provide.^118^ These results indicate that at least two of the green alternatives listed above have already been carried through to quantified sorption or mechanical performance, even though none has yet been benchmarked directly against an isocyanate-modified analogue under matched conditions.

Where comparable data exist, isocyanate chemistry retains a measurable edge in two respects. On hydrophobisation, the lignin-condensation route discussed above raised the water contact angle of the composite film only to around 57°, well below the values above 100° routinely achieved by isocyanate carbamation of NC with medium-to-long alkyl chains (Section 4.2.1);^118^ for applications such as oil–water separation, where a high contact angle is the operative design parameter, this places carbamation ahead of at least this green alternative. On grafting density, a silane-mediated route to polyol functionalisation of CNC achieved a maximum loading of only 0.32 mmol g^−1^, roughly a quarter of the 1.33 mmol g^−1^ of free isocyanate groups attainable by the standard TDI protocol (Section 4.2.3).^73^ These two comparisons should not be read as a general verdict in favour of isocyanate chemistry, since the green alternatives compared here were optimised for different objectives – wet-state mechanical integrity and matrix compatibilisation, respectively, rather than surface hydrophobicity or ligand density – but they indicate that isocyanate carbamation is not simply superseded by the available non-isocyanate routes and that the choice between them should be guided by which surface property is decisive for the intended application.

A systematic head-to-head comparison of the sorption performance of isocyanate-modified NC against these alternative chemistries for identical target pollutants represents a methodological gap already identified in Section 4.3.3, and remains an important direction for future research.

Despite regulatory pressure and the availability of green alternatives, NC isocyanate chemistry retains clear scientific and practical value in several well-defined scenarios. First, laboratory investigation of reaction mechanisms and structure–property relationships. The isocyanate route yields well-characterised model systems with known stoichiometry, controllable selectivity, accessible analytical methods (ATR-FTIR, ^13^C NMR, elemental analysis, and the quantification methodology described in ref. 92), and an extensive reference literature on related polyurethane systems. Selectivity is particularly well-defined in the TDI and IPDI cases. Without this foundational dataset, evaluation of NIPU alternatives would lack an adequate reference basis: NIPU-NC parameters must be benchmarked against reference isocyanate-NC materials, and that comparison is impossible without the latter.

This assessment sits in some tension with a recent comparative survey of cellulose surface-modification chemistries, in which urethanisation is ranked less favourably than several alternative routes on criteria such as reaction selectivity and linkage stability.^53^ The comparison in that survey is made across chemistries optimised for different purposes, however, and does not appear to weigh the specific combination of properties emphasised here – quantitatively defined site-selectivity, an extensive mechanistic reference literature, and a well-characterised route to an orthogonal reactive handle – all three of which remain, on the evidence reviewed in this section, more fully established for isocyanate chemistry than for any single alternative considered individually.

Second, industrial application in closed-process environments. When appropriate safety protocols and modern engineering controls are in place – closed systems, local exhaust ventilation, and reactors with automated reagent delivery – MDI- and HDI-based routes remain practically viable. The polyurethane industry successfully handles diisocyanates at scales of millions of tonnes per year under regulatory requirements far more stringent than those historically applied in academic chemistry; the same engineering controls are applicable to NC sorbent manufacturing. Third, high-performance niche applications where the specific properties of the urethane bond are irreplaceable. These include systems requiring the quantitative *para*/*ortho* selectivity of TDI or the site-selectivity of IPDI; materials with extreme thermal stability (≥250 °C) for thermal sorbent regeneration; and high-strength crosslinked networks that maintain fixed porosity under high-pressure column operation. NIPU analogues of these materials have not yet been reported, and their performance relative to isocyanate-based systems remains to be established experimentally.

The development of NC-NIPU sorbents represents a priority research direction for high-volume water treatment applications. This is particularly relevant for decentralised systems, point-of-use filters, and low-cost solutions in regions where regulatory infrastructure is limited, and where socioeconomic pressure favours cost reduction and process simplification. These materials should preserve the structurally useful features of isocyanate chemistry – covalent crosslinking, adjustable surface hydrophobicity, and multi-site sorption functionality – without the associated toxicological and regulatory risks. In conclusion, the role of NC isocyanate chemistry in water treatment is not reducible to a simple choice between adoption and rejection. A balanced development path encompasses: (i) retaining isocyanate chemistry as a fundamental research platform, particularly for mechanistic and comparative studies, with adherence to applicable safety standards; (ii) systematically shifting applied development towards less hazardous aliphatic analogues (HDI, IPDI) in preference to more toxic aromatic ones (TDI, MDI); (iii) intensively developing the NIPU direction as a green alternative capable of inheriting the structural advantages of urethane chemistry without its drawbacks; (iv) establishing quantitative reporting of residual NCO and total monomer content as standard practice; and (v) conducting direct head-to-head performance comparisons of isocyanate-NC, NIPU-NC, and silane-NC sorbents under identical experimental conditions to enable informed technology selection. Implementation of this agenda would enable the field to retain the methodological advantages of isocyanate chemistry while progressively reducing its dependence on regulated monomers.

## 5. Organic and inorganic pollutants in water

### 5.1. Synthetic dyes

One of the main environmentally hazardous compounds found in wastewater is organic dyes based on benzene and naphthalene, comprising a chromophore responsible for colour and an auxochrome that mediates attachment to the fibre.^119^ Synthetic dyes, with more than 100 000 different varieties, are widely used in various industries, including dyeing, textile, plastics, petroleum refining, and food processing. These dyes are highly water-soluble, provide bright colours, and are very resistant to fading, making them ideal for these applications.^120,121^ Annual dye production is 700 000 tons, of which approximately 300 000 tons are used in the textile industry. Approximately 10% of this total volume is discharged into wastewater.^119^

The most common type of dye that pollutes wastewater is azo dye. It accounts for 60% of all synthetic dye production. Even at low concentrations between 1.0 and 10.0 milligrams per litre (mg L^−1^), these substances are known to be teratogenic, carcinogenic, and mutagenic.^5^ For example, the cationic dye methylene blue, when present in concentrations above 4.7 micrograms per litre (µg L^−1^), has a chronic effect on aquatic organisms and can enter the human food chain without being noticed, leading to liver damage, chronic diseases, and haemolysis.^6,120^Table 5 summarises the main classes of synthetic dyes, their sources, and their effects on the environment and the human body.

**Table 5:** Effects of various dyes on the environment and the human body

Type of dye	Sources of water pollution	Permitted concentrations	Hazard to people and the environment	Reference
Azo dyes (Congo red, methyl orange)	Textile, leather, printing, and paper industries	1 mg L^−1^; dangerous even at low doses	Carcinogenic and mutagenic. It causes DNA damage, hypertension, blindness, gastritis, and liver damage	24 and 122
Cationic (basic) dyes (methylene blue, crystal violet)	Paper and textile industry, cosmetics and medicine	Above 4.7 µg L^−1^ for plankton	It causes methemoglobinemia, cyanosis, jaundice, seizures, corneal opacity, and tissue necrosis	1, 5, 6, 120, 122 and 123
Anthraquinone-based dyes (mordant violet, acid blue)	Textile and wool industry	0.05–0.1 mg dm^−3^ (depending on the specific substance)	Associated with the risk of bladder cancer, allergic reactions, and respiratory disorders	22 and 124
Sulfur and disperse dyes (direct blue 71, sulfur black)	Textile and leather industry	2 mg m^−3^	It causes genotoxic effects, allergic dermatitis, and mutations. It is capable of bioaccumulation in organisms. Even at low concentrations, it has an unpleasant odour	22 and 122
Phenolic compounds (chlorophenols, nitrophenols)	Oil refineries, paint production, pharmaceuticals, and pesticides	The limit for phenol in drinking water is less than 1 µg L^−1^	Affects the central nervous system, kidneys, and liver; causes muscle weakness, tremors, and respiratory arrest	8 and 22

### 5.2. Heavy metals

Wastewater often contains not only organic dyes but also complex mixtures of heavy metals, pharmaceutical compounds, pesticides, and other toxic components.^3,125^ Heavy metals such as aluminium (Al), copper (Cu), lead (Pb), mercury (Hg), iron (Fe), cobalt (Co), arsenic (As), and zinc (Zn) can bind with dye molecules and form various complex compounds. These compounds can have several harmful effects on the human body, including cancer, dementia, neurotoxicity, and organ failure.^119^ For example, lead ions are highly toxic to neurons and can impair cognitive function. Cadmium and mercury, on the other hand, are associated with the development of chronic diseases and have carcinogenic effects.^126^

The sources of major heavy metals entering water systems, their permissible concentrations, and the health hazards they pose are presented in Table 6.

**Table 6:** Characteristics of heavy metals and their permissible levels in wastewater

Heavy metal	Sources of water pollution	Water forms and complex types	Permitted concentrations	Harm to people and the environment	Reference
Lead (Pb)	Batteries, paints, glass and printing industry, pipes	Pb^2+^ ions in the form of complexes with EDTA and humic acids	10 µg L^−1^ (WHO)	Central nervous system, kidney, and liver damage, anemia, decreased children's IQ	7, 8, 23 and 126–128
Chromium (Cr)	Leather tanning, fertilizers, and the textile industry	Cr(iii) and highly toxic Cr(vi); complex compounds bound by citrates and EDTA	50 µg L^−1^ (WHO)	Carcinogen, causes lung cancer, allergic dermatitis, and gastrointestinal tract damage	5, 8, 120 and 126–128
Cadmium (Cd)	Mining, electroplating, batteries, phosphate fertilizer industries	Free Cd ions, chloride and organic complexes	3 µg L^−1^ (WHO)	Kidney failure, lung damage, bone fragility, and high organ toxicity	7, 8, 23, 123, 127 and 128
Mercury (Hg)	Electronics, chemical reagents, pharmaceuticals, coal processing	Inorganic and methylated forms (MeHg)	1 µg L^−1^ (WHO)	A potent neurotoxin that damages the brain (minamata disease), kidneys, and respiratory system	7, 8, 23, 121, 122 and 126–128
Copper (Cu)	Metallurgy, galvanising, pesticides, pipe corrosion	Cu^2+^ ions, citrate complexes, EDTA and ammonia complexes	2000 µg L^−1^ (WHO)	Liver and kidney damage, convulsions, mucous membrane irritation, diarrhea	1, 8, 122 and 126–128
Arsenic (As)	Pesticides, mining, metal smelting, fertilizer production	Arsenites (As(iii)) and arsenates (As(v))	10 µg L^−1^ (WHO)	Carcinogen, causes skin and liver tumors, vascular and nerve damage	8, 126, 128 and 129
Nickel (Ni)	Steel smelting, electroplating, battery manufacturing	EDTA, citrate, and ammonium complexes	20–70 µg L^−1^ (WHO)	Lung and sinus cancer, pulmonary fibrosis, dermatitis, and kidney failure	8, 122, 127 and 128
Zinc (Zn)	Electroplating, rubber manufacturing, agricultural runoff	Primarily unstable Zn^2+^ ions	3–5 mg L^−1^ (WHO)	Gastrointestinal cramps, anemia, lethargy, and metal fume fever	8, 122, 127 and 128

### 5.3. Limitations of traditional treatment methods

The choice of a pollutant removal method depends on the specific composition of the wastewater. Coagulation and flocculation are effective at removing suspended solids from water, including suspended solids, colloids, and organic dyes. However, these methods are not effective at removing micropollutants such as persistent organic dyes, heavy metal compounds, or pharmaceutical compounds.^130–132^ Electrochemical methods do not use large amounts of chemicals, making them environmentally friendly. However, they have some drawbacks, such as high energy consumption, electrode corrosion, and high equipment costs.^128,133^ Membrane microfilters can remove particles as small as 0.1 microns, but nanoscale ionic salts and metals may still be present in the effluent.^5,134,135^ Photocatalysis is distinguished by its ability to fully mineralise water, but it requires a powerful light source.^136,137^ Although biological treatment is relatively effective and environmentally friendly, it is difficult to completely remove heavy metals from the environment.^5,137^

They require expensive equipment and high energy consumption, and in addition, toxic waste products accumulate in water treatment equipment, which must then be disposed of in a safe and environmentally friendly way.^5,8^ These technological and economic limitations are driving increased scientific interest in adsorption, particularly in next-generation adsorbents based on nanocellulose.

It is within this context that the surface chemistry of the adsorbent becomes decisive: the capacity to introduce, with molecular precision, hydrophobic, chelating, or crosslinkable functionality determines selectivity, wet-state stability, and regenerability. Among the available routes, isocyanate-based carbamation offers a particularly versatile means of achieving such control, as analysed in detail in Section 4.

## 6. Nanocellulose-based adsorbents

### 6.1. General principles of adsorption

Adsorption is a common and effective method for removing pollutants from wastewater.^54,138^ Proper material selection can offer several advantages for adsorption, including ease of access, potentially low raw-material costs, large surface area, use across a wide range of pH values, and high effectiveness.^54,138^ During adsorption, pollutants are transferred from the liquid phase to the solid phase and become attached to the surface of the adsorbent. These processes are primarily driven by van der Waals forces, hydrogen bonding, ion exchange, electromagnetic interactions, and hydrophobic interactions.^54,138^

Nanocellulose-based materials have a high surface area, porous structure, and a significant number of hydroxyl and carboxyl groups. This permits the targeted introduction of amine, thiol, sulfonate, and other functional groups.^3,16,22,23^ Due to these properties, adsorbents based on nanocellulose can bind pollutants through various mechanisms, including electrostatic attraction, ion exchange, complex formation, hydrogen bonding, hydrophobic interactions, and π–π stacking.^16,29^ The active functional groups of nanocellulose and the main adsorption mechanisms are schematically shown in Fig. 3.

**Fig. 3:**
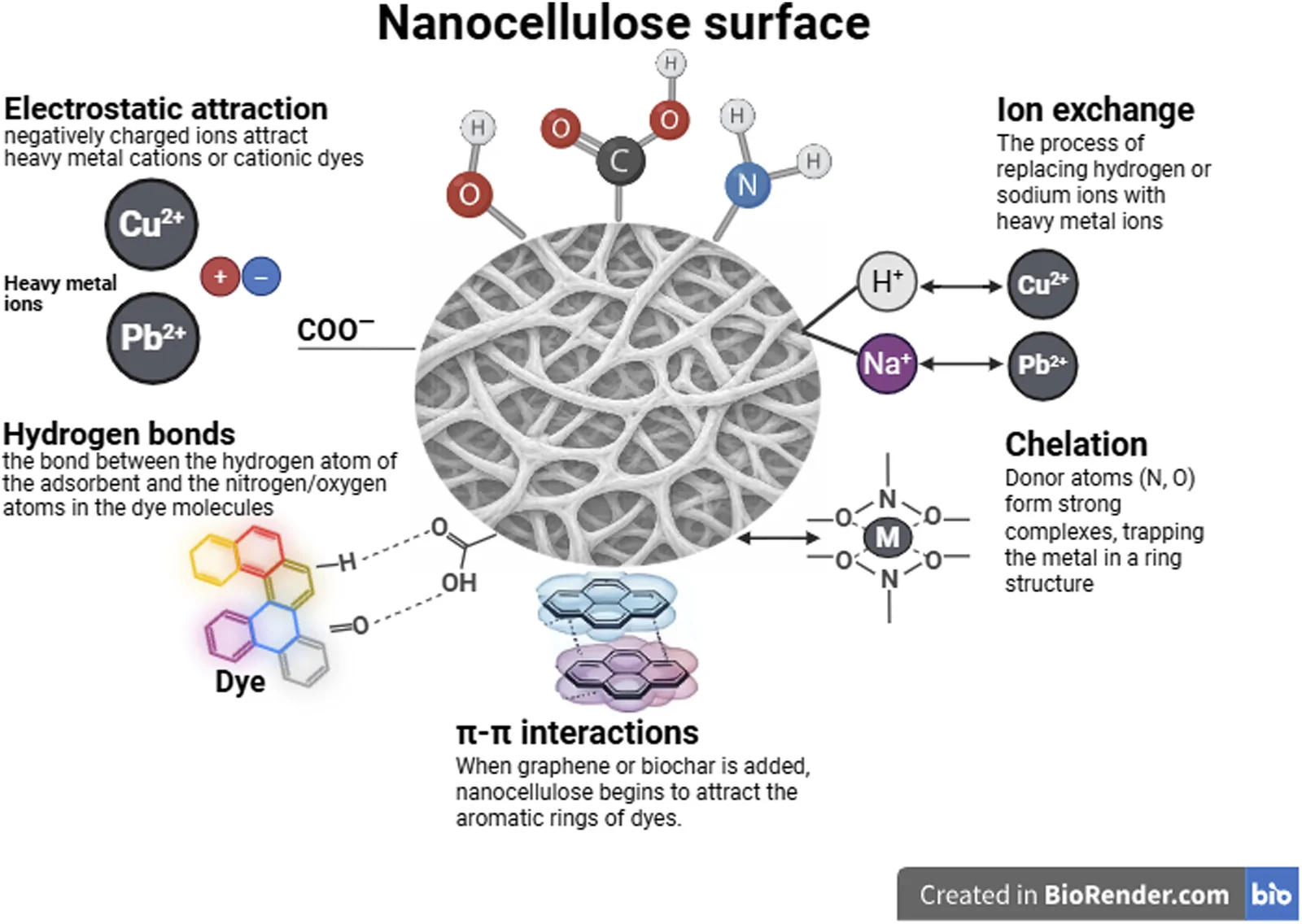
Active functional groups of nanocellulose (–OH, –COOH, –NH_2_) and their interaction mechanisms with pollutants: electrostatic attraction, ion exchange, coordination bond, hydrogen bonds, and π–π stacking (Created in BioRender).

#### 6.1.1. Mechanisms by pollutant class

The mechanism governing sorption is determined less by the nanocellulose scaffold itself than by the match between the charge and donor–acceptor character of the pollutant and the functional groups introduced onto its surface. This correspondence explains much of the three-order-of-magnitude spread in reported capacities across Tables 7 and 8.

**Table 7:** Effectiveness of nanocellulose-based hydrogels in adsorbing dyes

Adsorbent	NC component	Form	Dye	*q* (mg g^−1^)	pH	*C*_0_ (mg L^−1^)	Dose	*T*	Isotherm	Cycles	Ref.
Q-CNF aerogel, triisocyanate-crosslinked	CNF (cationic, TMA)	Aerogel	Red 180/blue CR19/orange 142	293/531/600 µmol g^−1^ (exp.); 314/557/627 (Langmuir)	7	—	200 mg/50 mL	25 °C	Langmuir	KCl elution, ×2	100
TOCN/PDMDAAC beads	TEMPO-CNF	Beads	MO/MB	404.86/520.83	7	—	—	298 K	Langmuir	—	139
TOCNF-3-APSA	TEMPO-CNF	Film	MB	526 (ungrafted: 370)	7	50–800	1 g L^−1^	RT	Langmuir	5 (91 → 74%)	140
BNCC–CMCT aerogel (Schiff base)	Hairy NCC	Aerogel	MB	785	7.5	240	0.2 g L^−1^	22 °C	Langmuir	6 (83.5%)	116
DA-C-CNF/ANF aerogel	Carboxymethylated CNF	Aerogel	Brilliant blue	67.93	4	5–300	15 mg/25 mL	—	Langmuir	3 (89%)	117
TEMPO-CNF/TiO_2_	TEMPO-CNF	Composite	Brilliant blue	162	7	25–600	1 g L^−1^	25 °C	Langmuir	—	141
T-CNF/SA nanocomposite	TEMPO-CNF	Composite	MB	216.4 (T-CNF alone: 185.2)	Neutral	—	—	25 °C	Langmuir	—	142
CMC/SA/60AH beads	NC from *Chlorella vulgaris*	Hydrogel beads	MB	109.03 (CMC/SA alone: 28.57)	—	10	0.08 g L^−1^	RT	Not fitted	—	143
TCNF/13.3% GO/PEI hydrogel	TEMPO-CNF	Hydrogel	MB	32.3	5.7	200	1.86 g L^−1^	25 °C	Not fitted	—	144
CMC/CNF–C aerogel (PEGDE)	Carboxylated CNF	Aerogel	MB	579.50 (exp.); 917.43 (Langmuir)	—	—	—	20–40 °C	Langmuir	5 (579.5 → 513.2)	145
CNF/PEI aerogel (CPE-2, ECH)	TEMPO-CNF	Aerogel	MO	1226 (pH 6); 1745 (pH 2)	6	50–1000	0.4 g L^−1^	RT	Langmuir	10 (>84%)	146
PDA-CNF-PEI aerogel	CNF	Aerogel	MO	265.9	4.41	200	—	RT	Langmuir	4 (89%)	91
Amf-CNF/LS aerogel	Amino-functionalised CNF	Aerogel	MB/RhB	171.23/148.81	8/6	1–500	1 g L^−1^	RT	Langmuir	—	86

**Table 8:** Efficiency of nanocellulose-based adsorbents in removing heavy metals

Adsorbent	NC component	Form	Metal	*q*_max_ (mg g^−1^)	pH	*C*_0_ (mg L^−1^)	Dose	*T*	Isotherm	Cycles	Ref.
APS(APTES)/MFC	MFC (∼50 nm)	Suspension	Ni/Cu/Cd	160/200/472	5	0.1–10 mmol L^−1^	3.33 g L^−1^	RT	Langmuir, Sips, D–R	HNO_3_/NaOH/EDTA	97
TOCN-PEI (glutaraldehyde)	TEMPO-CNF	Powder	Cu	52.32 ± 0.35 (RC 0.97; RC-PEI 2.11; TOCN 18.90)	5	20–100	1 g L^−1^	30 °C	Langmuir, nonlinear fit	1 M HCl	105
DA-C-CNF/ANF aerogel	Carboxymethylated CNF	Aerogel	Pb/Cr(vi)	78.80/41.77	5.8/2.5	5–300	15 mg/25 mL	—	Langmuir	3 (96%/80%)	117
TO-CTP aerogel (TMPTAP/PEI)	TEMPO-CNF	Aerogel	Cu	485.44	5.5	100	0.2 g L^−1^	25 °C	Langmuir	4 (>90%, EDTA-2Na)	90
CNF/TA/PVA aerogel	CNF	Aerogel	Pb/Cd/Cu	197/181/152 (exp. at *C*_0_ 100: 62.4/52.1/49.3)	6	0–120	0.05 g	60 °C	Langmuir	3 (>90%, EDTA)	147
CNF/polyacrylamide aerogel	Carbonylated CNF	Aerogel	Cu	240	—	—	—	—	Langmuir	**10 (>80%)**	148
CNC-g-P(AA/AM) aerogel	CNC (bamboo waste)	Aerogel	Pb	366.3 (pure CNC aerogel: 208.3)	4–6	200	1 g L^−1^	293 K	Langmuir	5 (81.3%)	149
PDA-CNF-PEI aerogel	CNF	Aerogel	Cu	103.5	5	1000	10 mg/20 mL	RT	Langmuir	4 (>91%)	91
Amf-CNF/LS aerogel	Amino-functionalised CNF	Aerogel	Cd	129.87	7	1–500	1 g L^−1^	RT	Langmuir	—	86
TEMPO-CNF aerogel	TEMPO-CNF	Aerogel	Cu	303.0	5	15–1000	0.013 g L^−1^	20 °C	Langmuir	—	87
TOCNF-Ag	TEMPO-CNF	—	Cd	116 (TOCNF alone: 78)	—	150	12 mg/15 mL	—	—	—	104
TCNF	TEMPO-CNF	—	Hg	178.6	6	500–1000	—	RT	—	—	78
CNF–2MPTMS sponge	TEMPO-CNF + MPTMS	Sponge	Hg	700 (CNF–1 MPTMS: 480)	6	50–700	0.5 g L^−1^	298 K	Langmuir	3 (0.1 M Na_2_EDTA)	50
ST-g-PAAc/CNW hydrogel	Cellulose nanowhiskers	Hydrogel	Pb	935.8 (without CNW: ∼12% lower)	—	—	25 mg	—	Chemisorption + intraparticle diffusion	6 (−8%)	150
FNH (titanate nanofibres/CDs)	CNC	Hydrogel	Cr(vi)	648.4 (predicted)	—	—	—	—	Langmuir	4 (83%)	151
BHA (TOCNF/PDChNF)	TEMPO-CNF + nanochitin	Aerogel	As(iii)	217	Neutral	—	—	—	—	—	152
ppy/NC	NC (H_2_SO_4_ hydrolysis)	Composite	Cr(vi)	560	2	50–300	15 mg/60 mL	—	Langmuir	6 (→70%)	153

Cationic dyes are bound principally through electrostatic attraction to carboxylate or sulfate ester groups, a mechanism that switches on above the p*K*_a_ of the carboxyl group (∼3–4) and accounts for the typical pH optimum of 6–9 observed for such systems. Anionic dyes, by contrast, are repelled by the same anionic surface and require the deliberate introduction of cationic centres – quaternary ammonium groups, polyethylenimine, or chitosan – before appreciable uptake occurs; a sulfonic-acid-grafted TEMPO-CNF, for example, achieved a 42% increase in methylene blue capacity relative to the ungrafted precursor, attributed to both increased anionic charge density and improved fibril packing.^140^

For metal cations, the operative mechanism is inner-sphere complexation rather than simple electrostatics, and selectivity follows a well-defined order established on TEMPO-oxidised cellulose: Pb^2+^ > La^3+^ > Al^3+^ > Cu^2+^ > Ba^2+^ > Ni^2+^ > Co^2+^ > Cd^2+^, Sr^2+^, Mn^2+^, Ca^2+^ > Mg^2+^, governed primarily by the electronegativity of the cation rather than by charge or ionic radius alone.^77^ This ordering rationalises the common design choice of thiol functionalisation for mercury and amine or polyamine functionalisation for lead and copper, since hydroxyl groups alone are comparatively weak ligands for these cations.^154^

Oxyanions such as Cr(vi), present as HCrO_4_^−^ or CrO_4_^2−^ depending on pH, require protonated amine or other anion-exchange centres, and their optimal pH is displaced toward acidic conditions – the mirror image of the behaviour observed for metal cations. In several systems, Cr(vi) removal additionally proceeds through partial reduction to the less toxic Cr(iii) at the sorbent surface, a mechanism confirmed spectroscopically in at least one polypyrrole–nanocellulose system.^153^

Oils and hydrophobic organic pollutants are captured by an entirely different mechanism – capillary imbibition and hydrophobic interaction within the pore network, governed by porosity and wettability rather than by the density of chemical functional groups. This is why uptake for such pollutants is reported in g g^−1^ rather than mg g^−1^, two to three orders of magnitude above the capacities typical of ionic sorbates.

#### 6.1.2. Influence of nanocellulose form

The form of nanocellulose used mainly determines the achievable density of subsequent functionalisation and the resulting particle architecture, rather than acting as an independent driver of sorption capacity. CNC, with crystallinity above 70%, exposes only its outer surface hydroxyls – around 15% of the total – to chemical modification, but offers high rigidity and a well-defined, monodisperse particle. CNF retains more accessible amorphous regions, forms entangled networks with high water retention, and is therefore the more common choice for hydrogel and aerogel architectures. BNC forms a lignin- and hemicellulose-free three-dimensional network directly during biosynthesis, without requiring subsequent assembly.

The relative contribution of nanostructuring itself, as distinct from surface functionalisation, has been quantified directly for Cu(ii) sorption on a polyethylenimine-grafted system: converting bulk regenerated cellulose to its TEMPO-oxidised nanofibrillar form increased the Langmuir capacity roughly 19.5-fold (from 0.97 to 18.90 mg g^−1^), whereas grafting PEI onto the bulk material achieved only a 2.2-fold increase, and grafting PEI onto the already-nanostructured material added a further 2.8-fold gain on top of that.^105^ This indicates that, at least for this class of sorbent, the transition to the nanoscale is the single largest contributor to overall capacity, with surface chemistry compounding rather than dominating the effect.

#### 6.1.3. Influence of adsorbent architecture

The same surface chemistry performs differently depending on the physical form into which the nanocellulose is assembled. Hydrogels offer high equilibrium capacity through swelling and volumetric access, but are limited by internal mass transfer and by poor mechanical integrity in the wet state, which complicates column operation. Membranes combine sorption with size-exclusion separation under applied pressure, where the limiting factor is typically the retention of structure and flux over extended operation rather than intrinsic capacity. Aerogels achieve the highest porosity (frequently exceeding 99%) and the highest volumetric capacity, but physically assembled networks are prone to collapse under repeated saturation–regeneration cycling.

The practical consequences of neglecting this architecture-dependence are illustrated starkly by a packed-column study in which unmodified TEMPO-oxidised CNF, rather than merely underperforming, swelled into a gel and blocked flow through the column entirely; functional operation was restored only after partial esterification reduced the material's water retention, at some cost to its carboxyl content and hence to its equilibrium capacity.^88^ A related trade-off appears between capacity and regenerability: sorbents engineered for very high uptake through dense functionalisation have also been reported to be markedly harder to regenerate than their more modestly functionalised counterparts, since the same strong binding that confers high capacity also raises the energetic cost of elution.^154^ Consistent with this, mechanical robustness conferred by covalent crosslinking has repeatedly proven to be a precondition for reliable operation rather than merely a desirable extra, and diisocyanate crosslinking is one route by which this robustness can be engineered directly into the sorbent architecture (Section 4.2.3).

#### 6.1.4. Why reported capacities are not directly comparable

Direct comparison of *q*_max_ values across Tables 7 and 8, or across the wider literature, is complicated by at least four independent methodological factors that are rarely controlled together.

First, apparent metal uptake at near-neutral pH can be confounded by precipitation of insoluble hydroxides that co-separate with the sorbent on filtration; without a matrix-matched blank lacking sorbent, part of the reported capacity may reflect precipitation rather than genuine adsorption, and this effect can be substantial even at metal concentrations below those typically used in sorption studies.^101^ Second, fitting isotherms by linearised regression, still the most common practice in this literature, can introduce errors of the order of tens of percent in the derived constants relative to nonlinear regression, and can mask the presence of more than one type of sorption site altogether.^102^ Third, the maximum capacity extracted from a Langmuir fit is itself sensitive to the concentration range over which the isotherm was measured: reported *q*_max_ values for the same material class have been shown to vary by more than an order of magnitude depending solely on the highest initial concentration tested, and are only meaningful as a true saturation capacity once the isotherm has genuinely reached a plateau.^103^ Fourth, even for chemically identical surface functionality, the sequence of processing steps – for instance, whether a crosslinking or complexing agent is introduced into a dilute dispersion or into an already-consolidated network – can determine whether the resulting linkages form predominantly within or between particles, with consequences for macroscopic properties that can differ by more than two orders of magnitude between otherwise similar preparations.^72^

Taken together, these four factors mean that a reported *q*_max_ value carries limited comparative meaning unless it is accompanied by the pH, initial concentration range, fitting method, and processing history under which it was obtained. Tables 7 and 8 report these parameters where available for precisely this reason; their frequent absence in the isocyanate-modified NC literature specifically is identified as a distinct gap in Section 4.3.3.

### 6.2. Hydrogels

Cellulose-based hydrogels are three-dimensional polymeric materials synthesised from cellulose derivatives, such as carboxymethyl cellulose, hydroxypropyl methyl cellulose, and ethyl cellulose. They play an important role in the adsorption process.^138,155^ The high porosity and the presence of numerous hydrophilic functional groups in hydrogels, such as –OH, –COOH, and –SO_3_H, allow them to absorb and retain a significant amount of water, while maintaining their structural integrity.^138,155^ The main strategies for crosslinking nanocellulose-based hydrogels are illustrated in Fig. 4. A three-dimensional network of interlaced nanofibrils forms a strong and flexible matrix through cross-linking. The hierarchical porosity of the material allows water molecules to move freely, increasing the number of adsorption sites. Covalent bridges and hydrogen bonds between the chains provide structural stability, preventing the material from dissolving during swelling (Fig. 4). The covalent bridges responsible for this wet-state stability are most commonly formed using citric acid, glutaraldehyde, or epichlorohydrin; diisocyanate crosslinking offers the same structural function while additionally modifying the polarity of the nanocellulose surface in a single step (Section 4.2.3).

**Fig. 4:**
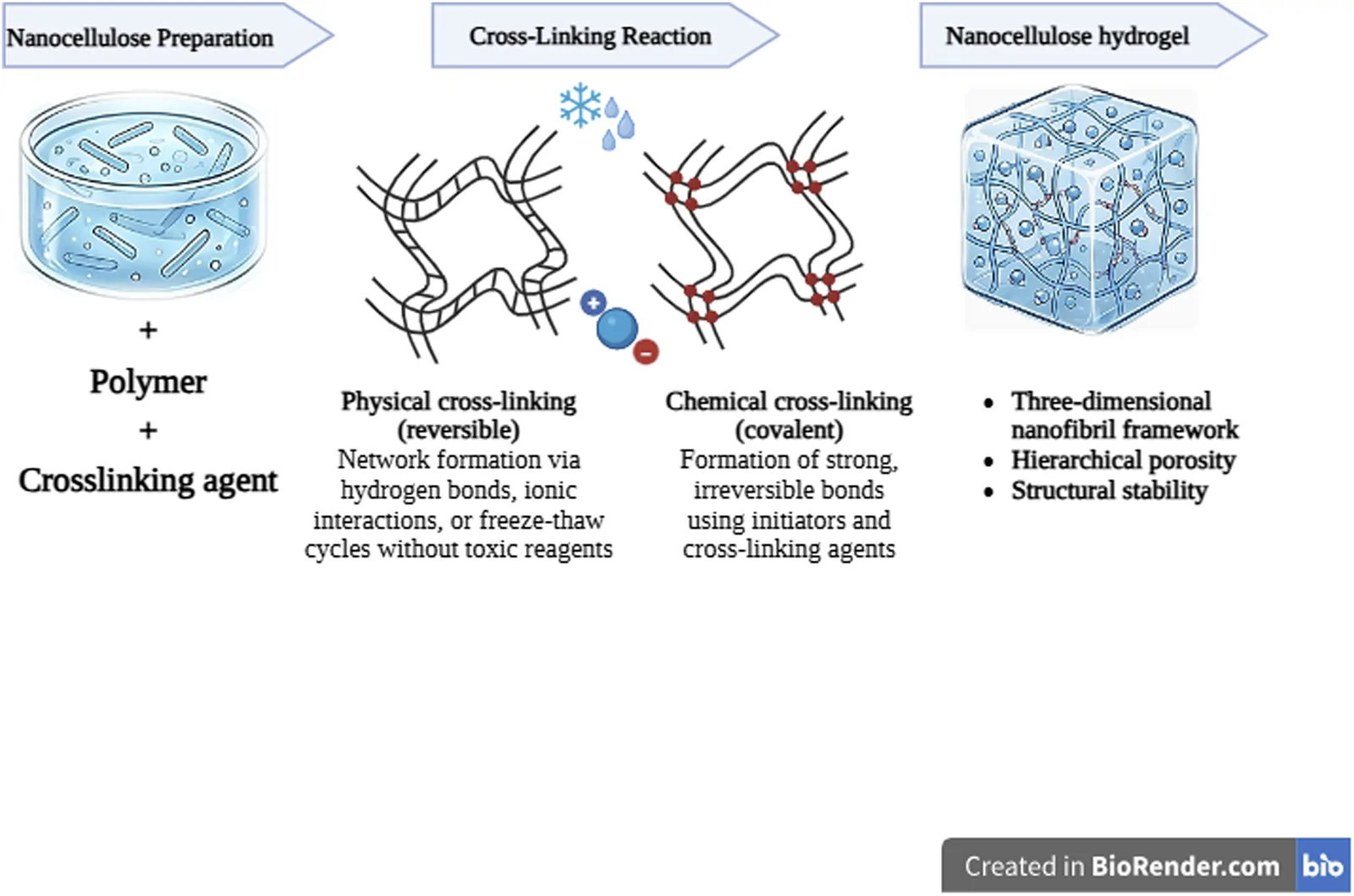
A schematic overview of the preparation of nanocellulose-based hydrogels (Created in BioRender).

Removal of synthetic dyes. Nanocellulose-based hydrogel systems have shown significant performance in removing synthetic dyes.^24^ An amphoteric TOCN-based hydrogel bead system, combining TEMPO-oxidised carboxylate groups with grafted quaternary ammonium groups, achieved Langmuir capacities of 404.86 mg g^−1^ for the anionic dye methyl orange and 520.83 mg g^−1^ for the cationic dye methylene blue at pH 7 and 298 K, with the amphoteric surface chemistry enabling effective removal of both dye classes by the same material.^140^ A related TEMPO-oxidised CNF system grafted with 3-aminopropyl sulfonic acid groups increased the methylene blue capacity from 370 to 526 mg g^−1^ relative to the unfunctionalised precursor, retaining 74% of its initial efficiency after five adsorption–desorption cycles.^140^ Chitosan-, carboxymethylcellulose-, and nanocellulose-based systems have been shown to be able to remove both cationic and anionic dyes. Approximately 99% of methylene blue and 82% of methyl orange were removed at pH 3. The efficiency was maintained even after 20 cycles of regeneration.^23^

Heavy metal removal using hydrogels based on carboxylated cellulose nanofibrils. These hydrogels have an adsorption capacity of approximately 171 mg g^−1^ for Pb^2+^, which removes approximately 90% of the metal. The values obtained for Cu^2+^, Ni^2+^, and Hg^2+^ were 182–230 mg g^−1^, 200 mg g^−1^ and 140 mg g^−1^ respectively.^126^ An SA/CMC-based hydrogel adsorbent modified with sulfuric acid-activated kaolin demonstrated values of 879.84 mg g^−1^ for Pb^2+^ and 543.50 mg g^−1^ for Cu^2+^. After five sorption–desorption cycles, approximately 80% of the initial efficiency was retained for lead and 79% for copper.^1^ The effectiveness of nanocellulose-based hydrogels in adsorbing organic dyes and heavy metals is summarised in Table 7.

### 6.3. Membranes

Nanocellulose-based membranes combine the properties of an adsorbent and a selective barrier, allowing them to remove pollutants through adsorption, size exclusion, and controlled filtration.^5,22^ A composite membrane based on sodium alginate, cellulose nanofibrils, and UiO-66 retains 99.9% of Pb^2+^ at 0.4 MPa and 98.5% and 96.5% of Cu^2+^ and Cd^2+^, respectively. The removal of Congo red, tartrazine, and methylene blue exceeds 98%.^5^ Furthermore, after ten cycles of filtration and regeneration, the membrane retained over 90% of its initial efficiency.^5^

CNC/CNF membranes are used for water filtration and deionisation processes, as well as for the separation of water–oil emulsions. Their high porosity and hydrophilic properties ensure efficient filtration with relatively low hydraulic resistance.

A related demonstration of the trade-off between packing density and permeability comes from phosphorylated TEMPO-oxidised CNF nanopapers prepared from aqueous *versus* ethanol suspensions: the aqueous-cast, densely packed nanopapers achieved a permeance of only 3–11 L h^−1^ m^−2^ MPa^−1^, whereas casting from ethanol produced a substantially more porous structure with a permeance exceeding 100 000 L h^−1^ m^−2^ MPa^−1^ – more than four orders of magnitude higher – at the cost of some mechanical strength.^89^ This illustrates that the processing conditions used to assemble a nanocellulose membrane can be as decisive for flux as the surface chemistry itself, and that the covalent crosslinking discussed for hydrogels and aerogels (Sections 4.2.3 and 6.2) offers one route to reconciling high porosity with the mechanical integrity needed for sustained filtration performance.

### 6.4. Aerogels

Aerogels made from nanofibres are characterised by a very low density (less than 2 mg cm^−3^) and ultrahigh porosity (>99%); their water retention capacity exceeds their own weight by hundreds of times (up to ≈100 g g^−1^).^156^ Nanofibril cellulose/graphene nanosheet nanocomposite aerogels showed an adsorption of 1178 mg g^−1^ for methylene blue and 585 mg g^−1^ for Congo red, while porous rGO/CNC structures have shown adsorption of up to 850 mg g^−1^ for dyes.^3,22^

Hydrophobised nanocellulose-based aerogels have also shown high efficiency in adsorbing petroleum products. They can adsorb up to 279 grams of castor oil per gram, 101 grams of diesel fuel per gram, and 185 grams of vegetable oil per gram. The removal efficiency of aqueous–organic mixtures has been shown to reach 99.97%.^22^

The mechanical fragility that limits many physically assembled NC aerogels can be overcome by covalent crosslinking; diisocyanate-crosslinked NC aerogels, discussed in Section 4.3.1, combine enhanced specific surface area, hydrophobicity, and repeated-compression durability within a single reaction step, illustrating the direct relevance of isocyanate chemistry to the adsorbent architectures described here.

### 6.5. Removal of other organic pollutants

Nanocellulose is also highly effective at removing other organic pollutants from wastewater, such as phenolic compounds, antibiotics, pesticides, and petroleum products. A composite material based on cellulose acetate modified with zinc oxide (ZnO) and magnetite was able to remove approximately 64% of phenol in two hours.^16^ Nanocellulose has been shown to effectively remove nitrates from agricultural wastewater and soil solutions, as well as certain pesticides such as chlorpyrifos, tetraconazole, diuron, difenoconazole, and nitenpyram.^16^ Regarding antibiotic removal, for ZIF-67/alginate, the adsorption capacity for ciprofloxacin was 364.89 mg g^−1^. However, for the CNT/GO/SA-based three-network systems, the capacity was found to be 158.5 mg g^−1^.^123^ Notably, none of the sorbents discussed above for PPCPs, pesticides, or petroleum-derived pollutants employs isocyanate chemistry, despite the fine-tuning of surface polarity offered by R-group selection during carbamation being, in principle, well suited to these targets (Section 4.3.3).

### 6.6. Sensing and monitoring systems

Nanocellulose-based hydrogels are being studied as potential environmental monitoring platforms. Fluorescent hydrogels made from nanocellulose, titanate nanofibrils, and carbon quantum dots can simultaneously detect and eliminate Cr(vi). The fluorescence of these hydrogels is quenched as the concentration of ions increases, providing a quick visual response. Similar systems using gold nanoclusters have shown high sensitivity to Hg^2+^. These studies suggest that the analytical and remediation functions could be integrated into a single material.^26^

### 6.7. Environmental factors affecting adsorption performance

The majority of the capacity data summarised in Tables 7 and 8 were obtained in single-component model solutions under optimised conditions. In real effluents, performance is governed by a set of environmental factors capable of altering the observed capacity by an order of magnitude. Four such factors and their consequences for nanocellulose sorbent design are considered below.

#### 6.7.1. Solution pH

pH is the single most influential parameter. It acts simultaneously on three independent characteristics of the system: the surface charge of the sorbent, the ionisation state of the pollutant, and the speciation of metal ions in solution.^154,157^

For carboxylate-bearing nanocellulose, the controlling value is the p*K*_a_ of the carboxyl group (∼3–4): below it the groups remain protonated and the electrostatic mechanism is suppressed; above it they are deprotonated and the surface acquires the negative charge required for binding metal cations and cationic dyes. In the acidic range, hydronium ions additionally compete directly with metal cations for the available surface sites, further reducing uptake.

The accessible range is bounded from above, however. At pH values above roughly 6–7, metal cations begin to hydrolyse and precipitate as hydroxides. The measured decrease in solution-phase metal concentration then includes a contribution unrelated to adsorption. This has been demonstrated directly for a nanocellulose–lignosulfonate aerogel, where a visible turbidity from Cd(OH)_2_ formation accompanied an apparent twentyfold rise in capacity between pH 6 and pH 10. The authors accordingly restricted their measurements to pH 7.^86^ Several other studies have likewise deliberately capped their working pH below the onset of hydroxide precipitation, and this practice should be regarded as a methodological requirement rather than an optional refinement (Section 6.1.4).

For oxyanions the dependence is inverted. Cr(vi) exists as HCrO_4_^−^ or CrO_4_^2−^ depending on pH, and its uptake requires a positively charged surface – that is, protonated amine groups – so that the optimum lies in the acidic region; removal efficiency for a polypyrrole–nanocellulose composite fell from 98% at pH 2 to around a third of that value at pH 8, as hydroxide ions began to compete with chromate for the same sites.^153^ A single material can therefore exhibit diametrically opposed pH dependences for cationic and anionic pollutants, which makes comparison of capacities measured at different pH values meaningless in principle.

This has direct bearing on the subject of this review. The hydrolytic stability of the anchoring linkage across the working pH range determines whether a sorbent is applicable to real effluents at all; the aromatic urethane linkage formed by TDI modification is not subject to the base-catalysed hydrolysis that limits silane-anchored analogues in alkaline media (Section 4.3.3).

#### 6.7.2. Ionic strength

Increasing ionic strength compresses the electrical double layer and screens electrostatic attraction between the sorbent surface and a charged sorbate. The effect is selective. Mechanisms based on electrostatic attraction and ion exchange are strongly suppressed, whereas inner-sphere complexation and chelation are affected far less, since they do not depend on long-range electrostatics.

Two contrasting cases from the nanocellulose literature illustrate this clearly. For an aerogel binding methylene blue predominantly through electrostatic interaction with carboxylate groups, addition of 0.1 M NaCl reduced the capacity from around 785 to 272 mg g^−1^ – a loss of nearly two thirds.^116^ By contrast, an amine-functionalised aerogel binding Cu(ii) through chelation retained 89.6% of its capacity in the presence of the same background electrolyte concentration. The authors took this as direct evidence that chelation, rather than electrostatics, dominates the binding.^90^

The practical implication is rarely stated explicitly: sorbents that rely on electrostatic attraction – TEMPO-oxidised and sulfated nanocellulose, or cationised systems for anionic dyes – are poorly suited to saline effluents, whereas materials bearing amine, thiol, or iminodiacetate ligands retain their performance. For nanocellulose sorbents intended for real industrial waters, this is an argument in favour of chelating functionalisation, of which the two-stage grafting route through a diisocyanate bridge is one implementation (Section 4.3.3).

#### 6.7.3. Competing ions and multicomponent systems

Real effluents contain a range of cations competing for a finite number of active sites. The competitive behaviour of a sorbent is conveniently expressed through the ratio of capacity in a mixture to capacity in a single-component system, values below unity indicating antagonism and values above unity synergism.^158^

Antagonism is the more common outcome. Systematic study of background electrolytes on a polypyrrole–nanocellulose composite showed Cr(vi) removal falling from 98% in their absence to 77% with NaCl, 72% with Ca^2+^, and as low as 54% with sulfate, the ordering reflecting differences in hydrated ionic radius and in affinity for the positively charged surface.^153^ Similar suppression has been documented across lignocellulosic sorbents generally, with binary and ternary systems consistently yielding lower capacities than single-metal controls.^158^ Of particular practical relevance are the hardness ions Ca^2+^ and Mg^2+^, present in natural and industrial waters at concentrations two to three orders of magnitude above those of target heavy metals.

Synergism, however, also occurs and should not be overlooked. For an amphoteric TOCN-based bead system, the presence of a second dye at higher concentration enhanced rather than suppressed uptake of the first,^139^ and comparable cooperative behaviour has been reported for biomass-based sorbents in dye–metal binary systems.^157^ Selectivity can also be preserved by design: a carboxymethylated CNF–amyloid fibril aerogel retained a distribution coefficient for Pb(ii) more than two orders of magnitude above those for Ca(ii) and Mg(ii) at equal concentrations, demonstrating that competition from hardness ions is not inevitable where the binding mechanism is sufficiently specific.^117^

The general principle is that competition is determined by differences in affinity rather than by concentration alone: where binding is purely electrostatic, abundant background ions compete effectively; where specific coordination dominates, selectivity is largely retained.

#### 6.7.4. Selectivity ordering and implications for design

Underlying these observations is a reproducible selectivity ordering, established directly on TEMPO-oxidised cellulose across the pH range 3–6 and largely independent of pH within it: Pb^2+^ > La^3+^ > Al^3+^ > Cu^2+^ > Ba^2+^ > Ni^2+^ > Co^2+^ > Cd^2+^, Sr^2+^, Mn^2+^, Ca^2+^ > Mg^2+^, with the electronegativity of the cation identified as the principal determining factor rather than charge or ionic radius.^77^ The position of Ca^2+^ and Mg^2+^ at the low-affinity end of this series explains why selectivity for target heavy metals over hardness ions is achievable at all on carboxylated surfaces, while the proximity of Cd^2+^ to Ca^2+^ in the same series indicates why cadmium is among the more difficult targets in hard waters.

Three requirements follow for future work on nanocellulose sorbents, including isocyanate-modified systems: evaluation in multicomponent solutions and real effluents alongside model systems; reporting of the full experimental conditions under which capacities were obtained; and explicit separation of adsorption from precipitation when operating above pH 6. None of these is currently met systematically in either the isocyanate or the silane branch of the literature, which is one reason a direct comparison of the two approaches remains unavailable (Section 4.3.3).

## 7. Advantages, disadvantages, and future research directions of nanocellulose-based adsorbents

### 7.1. Advantages

In recent years, nanocellulose-based materials have been recognised as promising and multifunctional materials for the production of more sustainable adsorbents used in the process of cleaning up contaminated environments and restoring ecosystems. This is due to their combination of physical, chemical, structural, and ecological properties, which ensure their high efficiency in removing pollutants of different classes from water systems.^3,16,22^

#### 7.1.1. Renewable raw materials and economic efficiency

Nanocellulose can be obtained from a wide range of biomass, including wood, agricultural residues, and other lignocellulosic resources.^20^ The use of agricultural waste, such as rice husks and straw, as a raw material for production may reduce raw-material costs, although a full techno-economic comparison with traditional adsorbents such as activated carbon is still lacking.^14,54,134^

#### 7.1.2. Biodegradability and environmental compatibility

Unlike many synthetic polymers, materials based on nanocellulose do not produce persistent residues.^3^ However, the environmental impact of chemically modified nanocellulose, including residual isocyanate monomers (Section 4.5), has not yet been fully assessed.

#### 7.1.3. High specific surface area and chemical activity

The high specific surface area of nanocellulose materials (hundreds of m^2^ g^−1^) provides numerous available sorption sites.^16,22,23,123^ The presence of functional groups such as –OH, –COOH, and –NH_2_ in molecules helps them to form chelate bonds with heavy metal ions. These bonds can participate in ion exchange reactions, form hydrogen bonds, and provide electrostatic attraction between the molecules and the ions.^16,54^

#### 7.1.4. Mechanical properties

Nanocellulose acts as a strong reinforcing agent in composite hydrogels, significantly increasing their strength and structural stability.^25,26,54^

#### 7.1.5. Regeneration capability

Adsorbents can be regenerated multiple times, up to 20 cycles in some cases. When using mild eluents, such as acidic or alkaline solutions, they maintain over 80–90% of their original efficiency.^23,54,123,134^

#### 7.1.6. Functionalisation potential

The use of various nanoscale capsules, such as metal oxide, carbon-based materials, and metal–organic frameworks, opens up the possibility of creating hybrid systems with enhanced sorption properties.^5,123^

The main advantages and disadvantages of nanocellulose adsorbents are summarised in Table 9.

Comparison of the advantages and disadvantages of nanocellulose

**Table:** 

Advantages	Description	Reference
Ecotoxicity	Nanocellulose is a renewable and biodegradable material derived from biomass. However, the ecotoxicity of nanoscale and, especially, chemically modified cellulose, including the potential effects of residual isocyanate monomers (Section 4.5), remains insufficiently studied	21, 25, 134, 159 and 160
Specific surface area	The material has a very high specific surface area (hundreds of m^2^ g^−1^), which creates numerous active sites for trapping pollutants. This provides high adsorption capacity	13 and 24
Chemical activity	The abundance of surface hydroxyl groups allows for easy chemical modification (carboxylation, amination, polymer grafting) to selectively remove certain ions or dyes	37, 160 and 161
Mechanical properties	Nanocellulose acts as a strong reinforcing agent in composite hydrogels, significantly enhancing their strength and structural stability	25 and 26
Economic efficiency	Using agricultural waste (rice husks, bran, straw) as raw material may reduce raw-material costs; a full techno-economic comparison with traditional adsorbents such as activated carbon is still lacking	14 and 134
Regeneration	Adsorbents can be regenerated multiple times (up to 20 cycles in some cases), and when using mild eluents (acidic or alkaline), they maintain over 80–90% efficiency	23 and 123

**Table:** 

Disadvantages	Description	Reference
Agglomeration	Due to their high surface energy and the formation of strong hydrogen bonds, nanoparticles tend to aggregate, which drastically reduces the available surface area	24 and 54
Scaling	Translation from laboratory to industrial scale remains problematic: batch-to-batch reproducibility of surface charge density and fibril dimensions is difficult to maintain, solvent-exchange steps required for many modification routes are impractical at volume, and drying methods that preserve porosity (freeze-drying, supercritical drying) are costly to scale	16 and 155
Energy consumption	Mechanical production methods (high-pressure homogenisation, grinding) require significant amounts of energy, which increases the cost of the product	14 and 16
Lixiviation	The leaching of nanoparticles or catalysts from the adsorption matrix into the treated water can lead to secondary contamination and requires a toxicity assessment	23 and 155
Moisture effect	When dried, nanocellulose may undergo a “hornification” process, losing its flexibility and rehydration ability; this irreversible collapse of the fibril network also reduces the accessibility of surface carboxylate groups, directly diminishing the number of sites available for subsequent sorption	24 and 87
Complex matrices	Most studies are conducted in model solutions containing a single pollutant; in real wastewater, competing ions can significantly reduce adsorption efficiency	123, 126 and 159

### 7.2. Future research directions

Overall, the analysis of the literature suggests that the environmental applications of nanocellulose are diverse and much more effective than simply adsorbing individual pollutants in wastewater.^159^ However, several scientific and technological gaps remain in this field.

The study of multicomponent systems is important. Detailed research into the adsorption processes of pollutants in real-world multicomponent systems is necessary, as most laboratory studies use model solutions.^155^ In real wastewater, competing ions can significantly reduce adsorption efficiency.^159^

#### 7.2.1. The production of multifunctional composite materials

The development of multifunctional nanocellulose based composites that integrate several water treatments processes.^123^ Hybrid systems containing metal–organic frameworks, carbon materials, or metal oxides have the potential to simultaneously adsorb pollutants, catalyse their degradation, and provide selective filtration. These systems can also enhance mechanical stability, making them more durable and reliable for use in various applications.

To achieve this, it is essential to develop stable and robust nanocellulose materials that retain their efficiency even after multiple regeneration cycles. This will ensure that the systems continue to function effectively over time, making them a valuable addition to environmental remediation efforts.^155^

#### 7.2.2. Deepening the isocyanate modification

Although isocyanate-functionalised nanocellulose adsorbents have demonstrated promising performance in removing heavy metals and organic dyes, there is still a lack of systematic studies in this area. In particular, investigating the adsorption behaviour of nanocellulose composites based on TDI, MDI, and HDI in real wastewater is an important future direction.^56^

#### 7.2.3. Industrial scaling up

Expanding production of nanocellulose-based adsorbents and developing efficient technologies for industrial application. In the future, the integration of nanocellulose materials into existing water treatment systems has the potential to significantly improve pollutant removal efficiency. This is a major challenge that must be addressed to ensure sustainable water treatment. The development of efficient technologies for the industrial production of these materials is essential.^24^

With respect to isocyanate chemistry specifically, three priorities emerge from the analysis in Section 4. First, quantitative sorption characterisation – adsorption capacity, isotherm constants, and thermodynamic parameters – is rarely reported for isocyanate-modified NC sorbents, which precludes meaningful comparison with the well-characterised TEMPO-, silane-, and phosphorylation-based systems. Second, no direct head-to-head comparisons exist between isocyanate-NC and silane-NC sorbents under identical conditions, despite the parallel development of the two fields. Third, isocyanate-NC sorbents remain essentially untested against the fastest-growing contaminant classes – microplastics, pharmaceuticals and personal-care products, and per- and polyfluoroalkyl substances – to which urethane chemistry is theoretically well suited. In parallel, the development of NIPU NC sorbents represents a priority green direction capable of inheriting the structural advantages of urethane chemistry – covalent crosslinking, tunable hydrophobicity, and multi-site sorption functionality – without the toxicological burden associated with free diisocyanate monomers.

## 8. Conclusion

This review has examined nanocellulose-based adsorbents for water remediation with isocyanate chemistry at its centre. The surrounding analysis establishes two points. NC sorbents deliver high capacities across dyes, heavy metals, oils and emerging organic pollutants. Surface functionalisation, rather than the choice of nanocellulose type, is the decisive performance lever. Against this background, four conclusions specific to isocyanate chemistry follow.

Among the routes compared here, diisocyanate carbamation is distinctive in delivering, within a single reaction step, covalent crosslinking, tunable surface hydrophobicity and an orthogonal reactive handle for subsequent ligand conjugation. Three particularly notable consequences distinguish this approach from the competing chemistries compared here: the quantitative site-selectivity available in TDI (93% *para*/*ortho*) and, under DBTDL catalysis, the near-quantitative selectivity of IPDI, which permits stoichiometrically predictable ligand loading; the free NCO loading of ∼1.3 mmol g^−1^ attainable in a single grafting step, roughly four times that reported for silane–polyol routes under comparable heterogeneous conditions; and the mechanical integrity of the covalent network, which preserves porosity through repeated saturation–regeneration cycles and under column pressure where physically assembled NC aerogels collapse or gel. The reaction is additionally atom-economical, generating no leaving groups or salt waste—a practical advantage where residual reagents could migrate into treated water.

Three limitations are equally clear. Incompatibility with aqueous media requires solvent exchange into an anhydrous medium, making the protocol harder to execute and to scale than aqueous TEMPO oxidation or moisture-tolerant silylation; blocked polyisocyanates offer a partial route around this constraint. The NCO loading attainable on crystalline NC imposes a ceiling on subsequent ligand density that is lower than the nitrogen content of the highest-performing polyamine comparator systems. The most commonly used aromatic diisocyanates are TDI and MDI. Both are respiratory sensitisers classified as suspected carcinogens. The regulatory burden falls on the residual monomer in the finished material, not on the chemically bound carbamate. On thermal stability, moreover, competing chemistries have been reported to deliver larger gains than carbamation. Reported improvements for isocyanate-modified NC are, in turn, confounded by sulfate removal and by sample-handling artefacts.

Four questions define the research agenda. First, quantitative sorption characterisation of isocyanate-modified NC remains essentially absent: the one system characterised with full isotherms employs the isocyanate as a structural crosslinker, with sorption governed by independently introduced cationic groups, so that isocyanate chemistry has not yet been established as the variable controlling performance. Second, no head-to-head comparison under identical conditions exists between isocyanate-NC and silane-NC sorbents, although such a study would require no new synthetic chemistry. Third, isocyanate-NC sorbents remain untested against the fastest-growing contaminant classes—microplastics, PPCPs and PFAS—to which urethane chemistry is theoretically well suited. Fourth, quantitative reporting of residual monomeric NCO should become standard practice, alongside the methodological safeguards identified in Section 4.3.3.

The role of isocyanate chemistry in NC sorbent design is not reducible to adoption or rejection. It retains clear value as a mechanistic research platform with well-defined stoichiometry and accessible analytics. The same is true in closed-process industrial settings, where the polyurethane industry's engineering controls already apply. For high-volume and decentralised water treatment, however, non-isocyanate polyurethane routes represent the priority direction. Cyclic carbonate aminolysis proceeds in aqueous or aqueous-alcoholic media. It avoids sensitisers entirely. The resulting polyhydroxyurethane backbone carries pendant hydroxyls, which constitute additional sorption sites. NC-NIPU systems have been synthesised and characterised thermomechanically. None, however, has yet been tested in water. This is the single most clearly defined opportunity identified by this review.

Nanocellulose-based adsorbents offer a further, more general advantage: they can potentially be produced from agricultural residues such as rice hulls, soybean hulls, and sesame hulls.^20^ This approach is consistent with the principles of sustainable development and the circular economy. Agricultural waste is converted into a functional material, and the associated environmental burden may be reduced, although life-cycle assessment data still needed.

## Author contributions

Yerkebulan Altynov: investigation, visualization, writing – original draft. Dana Ashiraliyeva: visualization, investigation, writing – original draft. Bibigul Rakhimova: formal analysis, writing – original draft. Aidyn Kazhmuratov: formal analysis, writing – original draft. Altynbek Kazez: formal analysis, writing – original draft. Kalampyr Bexeitova: validation, writing – original draft. Seitkhan Azat: validation, writing – review & editing. Kenes Kudaibergenov: conceptualization, funding acquisition, project administration, supervision, writing – review & editing.

## Conflicts of interest

There are no conflicts to declare.

## Data Availability

No primary research results, software, or code have been included in this review article, and no new data were generated or analysed as part of this review.
